# Antimicrobial resistance in urosepsis: outcomes from the multinational, multicenter global prevalence of infections in urology (GPIU) study 2003–2013

**DOI:** 10.1007/s00345-015-1722-1

**Published:** 2015-12-11

**Authors:** Zafer Tandoğdu, Ricardo Bartoletti, Tomasso Cai, Mete Çek, Magnus Grabe, Ekaterina Kulchavenya, Bela Köves, Vandana Menon, Kurt Naber, Tamara Perepanova, Peter Tenke, Björn Wullt, Truls Erik Bjerklund Johansen, Florian Wagenlehner

**Affiliations:** Northern Institute for Cancer Research, Paul O’Gorman Building, Medical School, Newcastle University, Framlington Place, Newcastle upon Tyne, NE2 4HH England, UK; Department of Urology, University of Florence, Florence, Italy; Department of Urology, Santa Maria Annunziata Hospital, Florence, Italy; Department of Urology, Trakya Medical School, Edirne, Turkey; Department of Urology, Lund University, Lund, Sweden; TB Research Institute, Novosibirsk, Russia; Jahn Ferenc South Pest Teaching Hospital, Budapest, Hungary; Cubist Pharmaceuticals Inc., Lexington, MA USA; Department of Urology, Technical University of Munich, Munich, Germany; S.R. Urology Institute, Moscow, Russia; Department of Microbiology, Immunology and Glycobiology, Lund University, Lund, Sweden; Department of Urology, Oslo University, Oslo, Norway; Department of Urology, Paediatric Urology and Andrology, Justus-Liebig-University, Giessen, Germany

**Keywords:** Urosepsis, Pathogens, Resistance, Prevalence

## Abstract

**Objective:**

Primary objective was to identify the (1) relationship of clinical severity of urosepsis with the pathogen spectrum and resistance and (2) appropriateness of using the pathogen spectrum and resistance rates of health-care-associated urinary tract infections (HAUTI) as representative of urosepsis. The secondary objective was to provide an overview of the pathogens and their resistance profile in patients with urosepsis.

**Population and Methods:**

A *point* prevalence study carried out in 70 countries (2003–2013). *Population studied included;* 408 individuals with microbiologically proven urosepsis, 1606 individuals with microbiological proof of HAUTI and 27,542 individuals hospitalised in urology wards. Main outcomes are pathogens and resistance identified in HAUTIs and urosepsis including its clinical severity. A statistical model that included demographic factors (study year, geographical location, hospital setting) was used for analysis.

**Results:**

Amongst urology practices, the prevalence of microbiologically proven HAUTI and urosepsis was 5.8 and 1.5 %, respectively. Frequent pathogens in urosepsis were *E. coli* (43 %), *Enterococcus spp.* (11 %), *P. aeruginosa* (10 %) and *Klebsiella spp*. (10 %). Resistance to commonly prescribed antibiotics was high and rates ranged from 8 % (imipenem) to 62 % (aminopenicillin/β lactamase inhibitors); 45 % of Enterobacteriaceae and 21 % of *P. aeruginosa* were multidrug-resistant. Resistance rates in urosepsis were higher than in other clinical diagnosis of HAUTI (Likelihood ratio <0.05).

**Conclusions:**

It is not appropriate to use the pathogen spectrum and resistance rates of other HAUTIs as representative of urosepsis to decide on empirical treatment of urosepsis. Resistance rates in urosepsis are high, and precautions should be made to avoid further increase.

**Electronic supplementary material:**

The online version of this article (doi:10.1007/s00345-015-1722-1) contains supplementary material, which is available to authorized users.

## Introduction

Sepsis is known to have high mortality rates (severe sepsis: 28 % and septic shock: 41 %) and urinary tract infections (UTI) are one of the leading causes (severe sepsis: 9 % and septic shock: 31 %) [1–4]. Rapid and appropriate management of sepsis, including the administration of an initially adequate intravenous antibacterial, is essential for optimal outcomes [1]. However, compared to other causes of sepsis, inadequate coverage was identified as an outstanding problem in urosepsis [5]. Therefore, evidence about the pathogen spectrum and antibacterial resistance especially in urosepsis needs to be well collected. Unfortunately, data on pathogen spectrum and resistance are usually derived from studies that investigated overall hospital acquired urinary tract infections (HAUTI), but not urosepsis [6]. Therefore, appropriateness of this approach needs questioning. Additionally, there is a lack of knowledge of pathogen spectrum and resistance in different clinical severity forms of sepsis that is associated with mortality outcomes [2, 3].

The primary aims of the present analysis were to identify (1) relationship of clinical severity of urosepsis with the pathogen spectrum and resistance and (2) appropriateness of using the pathogen spectrum and resistance rates of HAUTIs as representative of urosepsis. In addition, a timely description of the pathogens and their resistance profile in patients with urosepsis at urology departments was aimed at.

## Materials and methods

### The global prevalence of infections in urology (GPIU) study

GPIU study is a web-based multinational, multicentre point prevalence study performed annually on select days of November annually since 2003 on November of each year to investigate infections in hospitalised urological patients only. Each centre was allowed to join the study in only a single day every year from several time points within November. Participating centres provided information on hospital and urology ward characteristics and practice. At 8 a.m. of the selected study day, all urological patients presently hospitalised in the department were screened for the presence of HAUTI. The investigators have to state for each reported patient whether the infection is a HAUTI or not. Clinical diagnosis, individual characteristics, intervention characteristics, culture outcomes and antibiotic treatment of patients with HAUTI were documented. Clinical diagnosis criteria that also included criteria for sepsis severity were provided as part of the protocol to be used for clinical classification (eMethods). Standards used for urine cultures susceptibility assessment were noted (eTable-1). Data of the years 2003 and 2004 were combined in one group due to data file structure changes made in 2005 [7]. Study centres were categorised according to the geographical locations (Europe, Asia, South America and Africa) and hospital type (University, Teaching, District and others).

GPIU data from 2003 to 2013 were reviewed, and urosepsis (sepsis emerging through a UTI) patients with microbiological proof of infection were identified. A summary of cases included in the current analysis is provided in eFigure-2.

### Pathogens and susceptibility profile in urosepsis

#### Pathogens

All urine and blood cultures were analysed in the local laboratories according to their microbiological standards. Pathogen spectrum is listed in Table 1 and antimicrobial susceptibility of pathogens in Table 2. Pathogens with a frequency below 1 % were grouped as ‘others’.

**Table 1:** Pathogen spectrum in urosepsis, its subgroups (geographical location, sepsis severity) and other HAUTIs (MAGI, cystitis, pyelonephritis)

Pathogen	Geographical location				Sepsis severity^a,b^		Clinical diagnosis			Overall urosepsis
	Europe *n* (%)	Asia *n* (%)	Africa *n* (%)	Americas *n* (%)	Simple urosepsis *n* (%)	Severe urosepsis and uroseptic shock *n* (%)	MAGI *n* (%)	Pyelonephritis *n* (%)	Cystitis *n* (%)	
*Gram negative*										
*E. coli*	127 (41 %)	34 (52 %)	8 (38 %)	5 (50 %)	121 (46.9 %)	29 (37.2 %)	77 (37 %)	190 (45 %)	240 (43 %)	174 (43 %)
*Klebsiella spp.*	25 (8 %)	8 (11 %)	6 (29 %)	3 (30 %)	24 (9.3 %)	9 (11.5 %)	17 (8 %)	54 (13 %)	73 (13 %)	42 (10 %)
*P. aeruginosa*	39 (13 %)	3 (5 %)	0	0	24 (9.3 %)	11 (14. %1)	25 (12 %)	34 (8 %)	48 (9 %)	42 (10 %)
*Enterobacter* spp.	19 (6 %)	2 (3 %)	0	2 (20 %)	14 (5.4 %)	6 (7.7 %)	13 (6 %)	20 (5 %)	42 (7 %)	23 (6 %)
*Proteus spp.*	14 (4 %)	0	2 (10 %)	0	7 (2.7)	4 (5.1)	7 (3 %)	27 (6 %)	34 (6 %)	16 (4 %)
*Acinetobacter spp.*	2 (1 %)	5 (8 %)	0		3 (1.2 %)	3 (3.8 %)	1 (1 %)	6 (1 %)	9 (2 %)	7 (2 %)
*Citrobacter spp.*	3 (1 %)	1 (2 %)	0	0	2 (0.8 %)	0	2 (1 %)	7 (2 %)	8 (1 %)	4 (1 %)
*Gram positive*										
*Enterococcus.*	37 (12 %)	5 (8 %)	4 (19 %)	0	27 (10.5 %)	9 (11.5 %)	23 (11 %)	41 (10 %)	55 (10 %)	46 (11 %)
*S. aureus*	12 (4 %)	3 (5 %)	0	0	12 (4.7 %)	2 (2.6 %)	7 (3 %)	7 (2 %)	18 (3 %)	15 (4 %)
CoNS	8 (3 %)	1 (2 %)	0	0	7 (2.7 %)	0	11 (5 %)	6 (1 %)	9 (2 %)	9 (2 %)
Other gram (+) cocci	4 (1 %)	0	1 (5 %)	0	3 (1.2)	0 (0.0)	4 (2 %)	0	4 (1 %)	5 (1 %)
Other bacteria	13 (4 %)	0	0	0	9 (3.5 %)	3 (3.8 %)	18 (9 %)	21 (5 %)	17 (3 %)	13 (3 %)
Fungi	8 (3 %)	4 (6 %)	0	0	5 (1.9 %)	2 (2.6 %)	2 (1 %)	14 (3 %)	7 (1 %)	12 (3 %)
Total	311	66	21	10	258	78	207	427	564	408

^a^Severity of sepsis was included in the logistic regression model analysis that already included geographical region, study year and hospital type. Based on this model, severity of sepsis was found not to influence the pathogen spectrum (Likelihood ratio test [=0.342)

Geographical location was the only parameter to influence pathogen frequency (shown in previous steps)

^b^Sepsis severity was collected after 2007

**Table 2:** Resistance profile of antibiotics and antibiotic combinations in urosepsis, its subgroups (geographical location, sepsis severity) and other HAUTIs (MAGI, cystitis, pyelonephritis)

Pathogen	Geographical location				Sepsis severity^a^			Clinical diagnosis				Overall urosepsis
	Europe %(R/total)	Asia %(R/total)	Africa %(R/total)	Americas %(R/total)	Simple %(R/total)	Severe and shock %(R/total)	*p* ^**c**^	MAGI %(R/total)	Cystitis %(R/total)	Pyelonephritis %(R/total)	*p* ^b^	
Amx/BLI	58 % (100/172)	70 % (21/30)	92 % (11/12)	75 % (3/4)	60 % (82/136)	64 % (21/33)	NS	52 % (65/124)	46 % (160/345)	60 % (149/248)	0.003	62 % (135/218)
								NS	OR 0.5 (CI 0.3–0.8) *p* = 0.002	NS		
TZP	34 % (47/137)	40 % (10/25)	50 % (4/8)	67 % (2/3)	37 % (40/109)	35 % (12/34)	NS	26 % (26/99)	33 % (84/258)	30 % (57/189)	NS	36 % (63/173)
TMP/SMX	56 % (87/156)	50 % (15/30)	86 % (12/14)	63 % (5/8)	59 % (84/143)	61 % (22/36)	NS	54 % (63/117)	52 % (182/353)	53 % (122/232)	NS	57 % (119/208)
CIP	59 % (106/181)	61 % (22/36)	47 % (8/17)	22 % (2/9)	53 % (79/148)	55 % (23/42)	NS	49 % (76/155)	47 % (196/420)	49 % (157/324)	NS	57 % (138/243)
LVX	59 % (57/97)	57 % (4/7)	50 % (6/12)	67 % (2/3)	56 % (40/71)	63 % (12/19)	NS	42 % (41/98)	45 % (122/271)	39 % (76/196)	0.009	58 % (69/119)
								OR 0.4 (CI 0.2–0.7),* p* = 0.006	OR 0.5 (CI 0.3–0.9) *p* = 0.03	OR 0.4 (CI 0.2–0.7)* p* = 0.002		
CXM	57 % (78/137)	56 % (14/25)	71 % (10/14)	67 % (4/6)	60 % (68/113)	52 % (14/27)	NS	48 % (50/104)	42 % (142/339)	45 % (109/245)	0.008	58 % (106/182)
								NS	OR 0.5 (CI 0.3–0.7) *p* = 0.001	OR 0.5 (CI 0.3–0.8), *p* = 0.007		
CTX	52 % (77/147)	42 % (15/36)	31 % (5/16)	56 % (5/9)	50 % (68/135)	43 % (13/30)	NS	36 % (41/115)	36 % (130/363)	39 % (110/208)	0.02	49 % (102/208)
								OR 0.5 (CI 0.3–0.9) *p* = 0.03	OR 0.5 (CI = 0.3–0.8), *p* = 0.003	OR 0.6 (CI 0.4–0.9) *p* = 0.01		
CAZ	42 % (52/124)	71 % (17/24)	33 % (4/12)	67 % (2/3)	49 % (47/96)	27 % (8/29)	0.005	30 % (31/102)	35 % (114/326)	33 % (83/251)	0.008	46 % (75/163)
					(OR = 4.07,CI 1.45–11.44, *p* = 0.005)	–		OR 0.4 (CI 0.2–0.8), *p* = 0.01	OR 0.5 (CI 0.3–0.8),* p* = 0.005	OR 0.4 (CI 0.3–0.7), *p* = 0.002		
IPM	8 % (11/141)	13 % (4/32)	0 (0/7)	0 (0/6)	8 % (9/110)	7 % (3/43)	NS	12 %(14/114)	7 % (19/289)	12 % (29/252)	NS	8 % (15/186)
GEN	36 % (68/187)	46 % (21/46)	75 % (12/16)	44 % (4/9)	37 % (57/154)	42 % (23/55)	NS	42 % (63/150)	36 % (150/418)	40 % (130/326)	NS	40 % (105/258)
CAZ + CIP	38 % (42/111)	56 % (10/18)	33 % (4/12)	67 % (2/3)	45 % (37/83)	21 % (5/24)	0.004	25 % (23/93)	28 % (82/292)	27 % (61/227)	0.006	40 % (58/144)
					OR = 5.1 CI 1.5–17.5, *p* = 0.009)	–		OR 0.4 (CI 0.2–0.8),* p* = 0.009	OR 0.4 (CI 0.3–0.7) *p* = 0.003	OR 0.4 (CI 0.2–0.7) *p* = 0.001		
CAZ + GEN	30 % (29/97)	52 % (12/23)	25 % (3/12)	67 % (2/3)	38 % (30/80)	23 % (5/22)	NS	24 % (22/90)	24 % (67/281)	21 % (46/218)	0.025	34 %(46/135)
								NS	OR 0.5 CI 03–0.9, *p* = 0.02	OR 0.5 CI 0.2–0.7, *p* = 0.003		
CAZ + TMP/SMX	30 % (22/74)	50 % (8/16)	25 % (3/12)	67 % (2/3)	41 % (29/71)	6 % (1/18)	0.000	23 % (16/70)	25 % (58/232)	24 % (38/158)	NS	33 % (35/105)
					OR 24.9 CI 2.4–255, *p* = 0.007	–						
TZP + CIP	33 % (36/108)	32 % (6/19)	50 % (4/8)	67 % (2/3)	36 % (30/83)	39 % (10/26)	NS	20 % (17/87)	27 % (63/231)	20 % (33/165)	0.03	35 % (48/138)
								OR 0.4 (CI 0.2–0.9) *p* = 0.03	NS	OR 0.5 (CI 0.3–0.9) *p* = 0.04		
TZP + GEN	20 % (19/93)	26 % (6/23)	50 % (4/8)	67 % (2/3)	26 % (20/77)	19 % (5/26)	NS	13 % (11/84)	18 % (29/161)	21 % (49/231)	NS	24 % (31/127)
TZP + TMP/SMX	20 % (14/69)	36 % (5/14)	50 % (4/8)	67 % (2/3)	30 % (19/64)	19 % (3/16)	NS	16 % (11/69)	22 % (44/199)	19 % (24/129)	NS	26 % (25/94)
CIP + GEN	31 % (41/134)	44 % (14/32)	44 % (7/16)	25 % (2/8)	31 % (35/114)	35 % (11/32)	NS	32 % (39/124)	32 % (114/358)	32 % (85/268)	NS	34 % (64/190)
CIP + TMP/SMX	37 % (37/102)	42 % (10/24)	50 % (7/14)	25 % (2/8)	40 % (40/99)	25 % (6/24)	NS	30 % (28/95)	33 % (101/302)	33 % (63/190)	NS	38 % (56/148)

*Amp/BLI* ampicillin or amoxicillin/BLI, *TZP* piperacillin/tazobactam, *CIP* ciprofloxacin, *LVX* levofloxacin, *CXM* cefuroxime, *CTX* cefotaxime, *CAZ* ceftazidime, *IPM* imipenem, *GEN* gentamicin, *TMP/SMX* trimethoprim/sulfamethoxazole

^a^Severity of sepsis was included in the logistic regression model analysis that already included geographical region, study year and hospital type. Based on this model resistance rates of CAZ and two of its combinations (CAZ + TMP/SMX and CAZ + CIP) showed a lower resistance rate in simple urosepsis. However, the wide confidence intervals are of notice

^b^Multiple logistic regression analysis, Likelihood ratio *p* value

#### Resistance profile

Resistance rates were determined for 10 antibiotics and eight antibiotic combinations (Table 2). These were registered as resistant, intermediate and sensitive. For analysis intermediate and resistant isolates were grouped together.

Frequently used antibiotics in urology were identified from previous publications of the GPIU [8]. Combinations of these that do not have a common resistance mechanism were analysed for resistance. These were: ceftazidime and piperacillin/tazobactam each combined with ciprofloxacin, gentamicin or TMP/SMX, and the combinations ciprofloxacin and gentamicin or TMP/SMX. This was done by combining the resistance categories and obtaining a three-tier grouping as: (1) resistant to both agents, (2) resistant to single agent and (3) sensitive to both agents.

#### MDR pathogens

Multidrug-resistant bacteria classification was carried out according to the ECDC and CDC definitions of multidrug (MDR), extensive drug (XDR) and pan-drug resistance (PDR) [9]. For this purpose, pathogens were classified as Enterobacteriaceae, *P. aeruginosa* and others. Some antibiotics needed for a complete ECDC resistance classification (teicoplanin, telavancin, tigecycline, daptomycin, chloramphenicol, moxifloxacin and streptogramins) were absent in the GPIU.

### Comparison of clinical severity of urosepsis

Data on severity of urosepsis were collected from 2007 onwards using the following classification: simple urosepsis, severe urosepsis, and uroseptic shock [10–12]. Severe sepsis and septic shock were grouped together and compared with simple urosepsis.

### Appropriateness of using HAUTIs as representative of urosepsis

All patients within the GPIU study were stratified according to the clinical diagnosis. These were: asymptomatic bacteriuria (ASB), male accessory gland infection (MAGI), cystitis, pyelonephritis and urosepsis. Comparisons were made except for ASB as it is not regarded as an infection [13].

### Statistical analysis

Study data were imported from the web-based portal into SPSS v.20.0 (SPSS Inc., Chicago, IL, USA) for analysis.

Bacterial spectrum and resistance rates were the primary outcome. Categorical data were compared with the Chi-square test. A logistic regression model was generated to determine parameters that influence studied outcomes. Baseline parameters included in the model were: hospital type, geographical location and study year (e-figure 3). Urosepsis severity and type of non-urosepsis HAUTI (excluding ASB) were added in the baseline model separately. Parameters that influence the outcomes significantly were identified with the Likelihood ratio test at a significance level of 95 %. Identified parameter magnitude and direction of influence on outcomes are presented as odds ratios (OR) with confidence intervals (CI) at a 95 % level of significance.

## Results

### Prevalence of urosepsis

Diagnosis of HAUTI was made in 2107 (7.7 %) cases amongst 27,542 patients screened. Microbiological proof of infection was available in 1606 (5.8 %) cases. In total, 408 patients had microbiologically proven urosepsis (25.4 % of HAUTIs; 1.5 % of total study day population)(e-Figure 2).

Cases were registered from Europe (*n*:311–76 %), Asia (*n*:66–16.1 %), Africa (*n*:21–5.1 %) and USA (*n*:10–2.4 %). Type of hospital cases were registered from university (*n*:228–56 %), teaching (*n*:107–26 %), district (*n*:69–17 %) and others (*n*:4–1 %). Mean age of patients with urosepsis was 63 ± 17 years, and the female-to-male ratio was 3:7. Mean Charlson comorbidity score was 2.48 ± 2.61 [14]. An intervention prior to the episode of urosepsis was reported in 324 (79 %) cases (clean: *n*:77–24 %, clean contaminated: *n*:99–31 %, clean contaminated with bowel segments opened: *n*:57–18 %, contaminated: *n*:28–9 %, infected: *n*:63–19 %, missing: *n*:2). A urinary catheter at the time of diagnosis was present in 287 (70 %) cases, and urinary tract obstruction was reported in 234 (57 %) cases. Urolithiasis was reported in 20 % (*n*:76, missing *n*:21) of cases.

Simple sepsis was seen in 258 (77 %) and severe sepsis/septic shock in 78 (23 %) patients. Remaining 72 patients were not classified for severity of sepsis.

### Pathogens and susceptibility profile in urosepsis

#### Pathogens

Pathogen order from most frequent to least is as follows: *E. coli* > *Enterococcus* spp. > *P. aeruginosa* > *Klebsiella* spp. > *Enterobacter spp.* > *Proteus* spp. > *S. aureus* > *Candida* spp. > *CoNS* > *Acinetobacter* spp. > *Citrobacter* spp. (Table 1).

The pathogen spectrum showed annual fluctuations, but this was not accompanied by an overall trend of change (Likelihood ratio *p* > 0.05) (e-Figure-4). Geographical location was the only parameter to influence pathogen spectrum (Likelihood ratio *p* = 0.001) (Table 1).

#### Resistance profile of pathogens

Overall resistance rates were lowest for imipenem (8 %), while for all other remaining antibiotics, resistance ranged from 36 to 62 % (Table 2). Geographical variation in the resistance rates of ampicillin/BLI (Likelihood ratio *p* = 0.05), gentamicine (Likelihood ratio *p* = 0.01) and piperacillin/tazobactam + gentamicin (Likelihood ratio *p* = 0.03) was statistically significant (Table 2). Annual fluctuation in the resistance rates was not statistically significant (Likelihood ratio *p* > 0.05) (e-Figure-4).

#### MDR pathogens

MDR rates for *Enterobacteriaceae* (*n*:259–63.4 %) was 45 %, and for *P. aeruginosa* (*n*:42–10.3 %) it was 21 %. Remaining rare pathogen subgroups (*n*:107–26.2 %) were not classified for MDR due to insufficient numbers to carry out analysis. Further categorisation into XDR etc. was not performed due to missing full antibiotic susceptibility testing against antibiotics.

### Comparison of clinical severity of urosepsis

Bacterial spectrum did not differ according to severity (Likelihood ratio test *p* = 0.34) (Table 1). Resistance rates for single agents in simple and severe urosepsis ranged from 8 to 60 and 7 to 64 %, respectively (Table 2). Rates of resistance for ceftazidime and two of its combinations tested (large confidence intervals) were significantly higher in simple sepsis (multiple logistic regression analysis.

### Appropriateness of using HAUTIs as representative of urosepsis

Clinical diagnosis of HAUTI with microbiological proof were as follows: pyelonephritis (*n*:427–27 %), cystitis (*n*:564–35 %) and MAGI (*n*:207–13 %). Pathogen distribution varied according to clinical diagnosis (Likelihood ratio test *p* = 0.012) (Table 1).

Highest resistance rates against all antibiotics and antibiotic combinations were observed in urosepsis compared with other HAUTIs (Table 2). These were statistically significant for ampicillin + BLI, levofloxacin, cefuroxime, cefotaxime, ceftazidime, ceftazidime + gentamicin, ceftazidime + ciprofloxacin and piperacillin/tazobactam + ciprofloxacin (logistic regression model) (Table 2).

## Discussion

### Prevalence of urosepsis

Urosepsis prevalence in urology patients was 1.5 %, and a quarter of patients with HAUTI were diagnosed with urosepsis. There are no directly comparable studies looking at urology patients in specific. Urosepsis rates in intensive care units (ICU) amongst patients with infections from a study conducted in Germany were 30.8 % (severe and shock) [15]. Another study reporting on nosocomial UTIs treated by non-urological departments identified the rate of severe urosepsis and uroseptic shock as 2 and 0.3 %, respectively [16].

### Pathogens and susceptibility profile in urosepsis

Annual pathogen spectrum was similar throughout the 11-year study time frame. Gram negatives contributed to approximately 75 %. This is different than reports of overall sepsis showing Gram positives (52 %) as the leading pathogen followed by Gram negatives (37 %) [2]. In the GPIU study pathogen spectrum differed with geographical region, previously shown in the SENTRY study also [17]. However, the published SENTRY results did not provide details on the clinical diagnosis of UTIs.

The presented antibiotic resistance rates are of great concern. The only antibiotic group with a resistance rate below 10 % were carbapenems, which is comparable to other HAUTIs we previously reported [18]. Remaining other antibiotic group resistance was above 36 %. Given the fact that for empiric treatment of severe infections resistance rates should not be higher than 10 %, we have limited options in urosepsis [11].

Fluctuations of annual resistance rates in urosepsis were not accompanied by a time trend of change within the study years. This finding seems counterintuitive as most studies report a concern of increase in resistance [6, 19]. However, resistance rates in our study are higher than the previous reports. For instance, a study from the UK with a cohort from urology patients including all HAUTIs reported a resistance rate below 25 % for *E. coli* to ciprofloxacin, which is almost half the rate we identified at baseline 2003/4 [6]. Capture of a meaningful change in our cohort with already high rate of resistance would require substantial changes. Therefore, the current results show that resistance rates in urology practice of urosepsis are already high.

One strategy to mitigate high resistance rates for empirical treatment can be the use of combination treatment of different antibiotic classes, until susceptibility results allow test specific treatment. Overall resistance rates for combination of antibiotics ranged from 24 to 40 %. Combinations showed resistance rates less than most single agents and not influenced from location or time. Resistance of single agents was published by ECDC in 2013 was similar to the GPIU while combination rates were markedly lower in ECDC [20]. However, the ECDC report is not directly comparable with the GPIU as it is not representative of urological patients. Further studies of combination agents in urosepsis are needed.

Recent reports of MDR pathogens are of significant concern both for hospital and community acquired infections [21, 22]. However, lack of a universal clinical definition for MDR in these studies limits interpretations. In the current analysis, we attempted to use the latest definition by ECDC/CDC of MDR to provide coherent results [9]. The GPIU registry started prior this definition, and we lacked some of the antibiotics required in their definitions limiting our ability of an accurate classification. Nevertheless, considering MDR pathogens are resistant to one or more antibiotics in two or more antibiotic categories [9], the scale of the problem becomes more apparent. The new MDR classification was easily identified in our data. Future study designs that include CDC/ECDC definitions of MDR would provide coherent comparison amongst different studies.

Resistance rates were lowest in Europe where a statistically significant difference was observed with ceftazidime, gentamicin and ampicillin + BLI. In our previously published analysis, the antibiotic consumption in regions seemed to overlap with the resistance rates identified in the current analysis [23]. As expected, geographical areas with higher consumption of antibiotics do also have higher resistance rates in urosepsis.

### Comparison of clinical severity of urosepsis

Arguably due to higher rate of mortality [2, 3] and resource consumption [24–26], more attention has been paid to severe sepsis than to simple. Within the GPIU cohort, three quarter of urosepsis patients were simple cases that may shift to a more severe vignette if not managed appropriately. In the current analysis, resistance was not associated with severity. The only exception for this was a statistically significant lower resistance rate in severe cases for ceftazidime, ceftazidime + TMP/SMX and ceftazidime + ciprofloxacin. However, the wide confidence intervals are of attention and severity of sepsis based antimicrobial differences should be confirmed with further studies. Until more data emerges, sepsis severity should not be a parameter to influence empirical antimicrobial treatment decisions.

### Appropriateness of using HAUTIs as representative of urosepsis

Evidence for urosepsis pathogen spectrum and resistance is gathered from complicated UTI reports [16, 17] and local surveillance data [6]. The current analysis identified that this approach is not appropriate as the pathogen spectrum and resistance are different in urosepsis compared to other HAUTIs. For instance, ceftazidime and levofloxacin, one of the recommended first-line treatments in urosepsis, [11] showed higher resistance in urosepsis than other HAUTIs (Table 2). Additionally, other antibiotics such as amoxicillin + BLI, cefotaxime, cefuroxime, ceftazidime combined with ciprofloxacin or gentamicin and piperacillin/tazobactam combined with ciprofloxacin had a significantly higher rate of resistance in urosepsis compared with other HAUTIs. In summary, the current analysis has shown that the pathogen spectrum and resistance rates in urosepsis are different than other HAUTIs.

### Limitations of the current analysis

Lack of coordinated surveillance studies was pointed out by the World Health Organization as a major gap in the topic of global antimicrobial resistance [20]. Although the GPIU study is a coordinated global HAUTI surveillance study of urology patients, certain geographical areas are not well represented contributing to limitations of the current analysis. In particular, lack of larger data from Africa and USA makes it hard to draw conclusions for these regions.

The GPIU study is focused on urological hospitalised patients only. While this is a powerful domain, it may raise two issues: Firstly; it is highly likely that urosepsis patients managed in ICUs by other specialities have been missed. Therefore, we did not report on overall hospital prevalence and instead have reported prevalence in urology departments. Secondly, participating centres may be more actively involved in infection control with a higher tendency of participating in surveillance studies. Therefore, the outcomes obtained should be approached cautiously and be regarded as indicators of the situation amongst urology practice. Moreover, these indicators should not be solely used as a recommendation for antibiotic treatment of HAUTIs, but as an indicator that needs to be tailored to the local situation.

## Conclusion

It is not appropriate to use the pathogen spectrum and resistance of other clinical diagnosis of HAUTIs as representative of urosepsis. In addition, the geographical variability of resistance rates makes it essential to have local surveillance reports on urosepsis separate from other HAUTIs in determining the appropriate empirical treatment. Adoption of the CDC/ECDC definitions of MDR for future epidemiological studies is necessary.

## Electronic supplementary material

Below is the link to the electronic supplementary material.

Supplementary material 1 (PDF 2310 kb)
