# Supplementary material for: Antimicrobial resistance in urosepsis: outcomes from the multinational, multicenter global prevalence of infections in urology (GPIU) study 2003–2013
Source: World J Urol. 2015 Dec 11;34:1193–200. doi: 10.1007/s00345-015-1722-1 (PMC4958125; doi:10.1007/s00345-015-1722-1)
Supplement: Supplementary file 1 — Supplementary material 1 (PDF 2310 kb) [file 345_2015_1722_MOESM1_ESM.pdf]

1. Figures 2
2. Tables 7
3. CLASSIFICATION OF UTIS Within the GPIU Study 8
3.1. Introduction 8
3.2. Level of infection 8
3.3. Grade of severity 8
3.4. Pathogens 11
3.5. UTI Classification 11

1. ***Figures***

eFigure 1; GPIU Study conduct summary

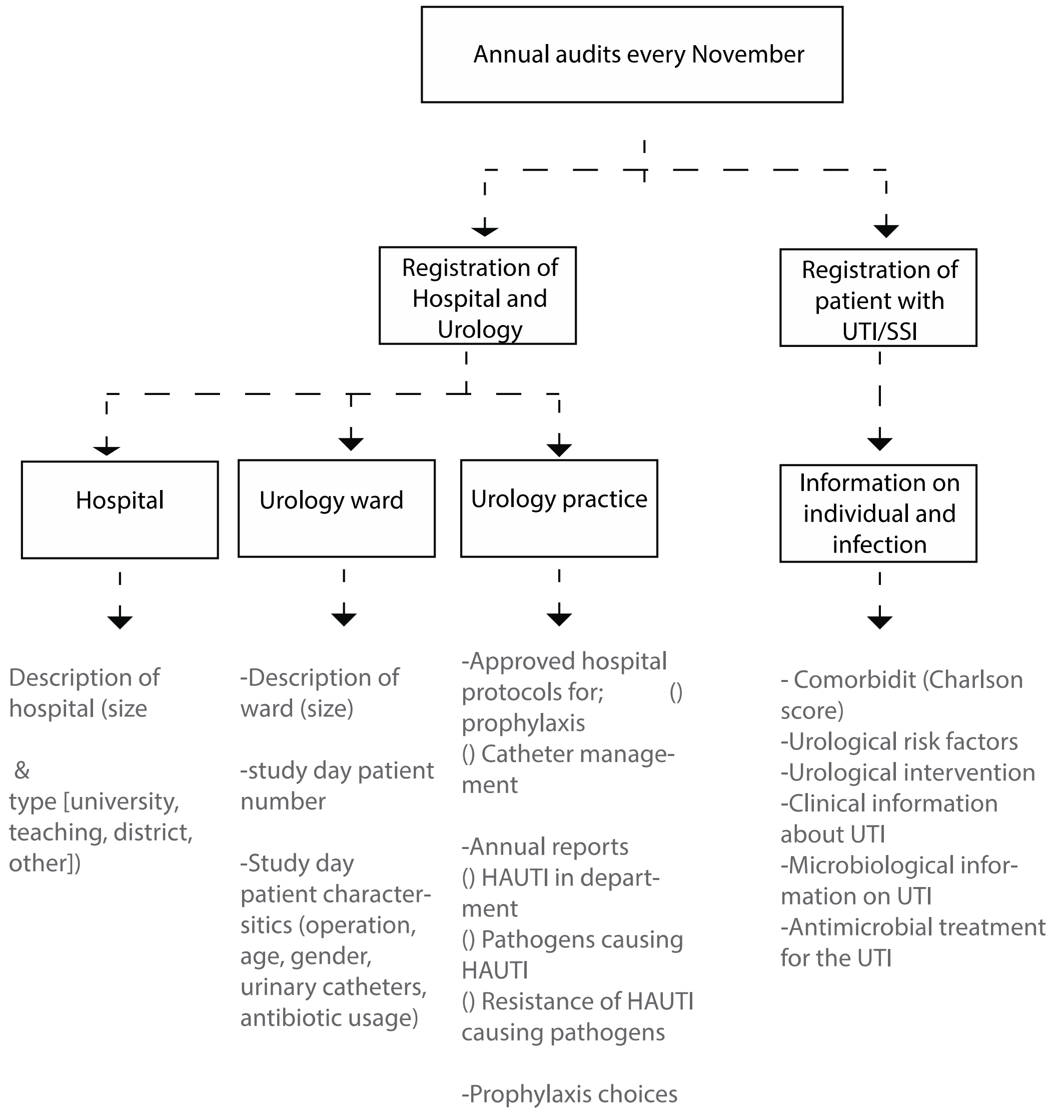

eFigure 2. Patient/ case disposition.

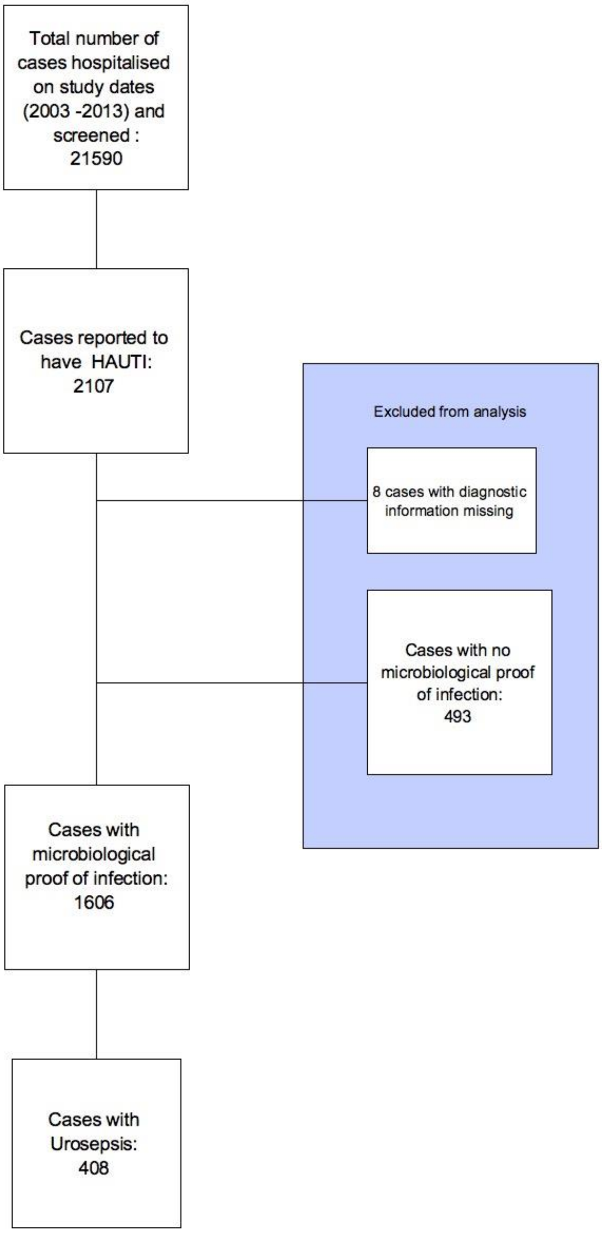

eFigure 3; Statistical model structure.

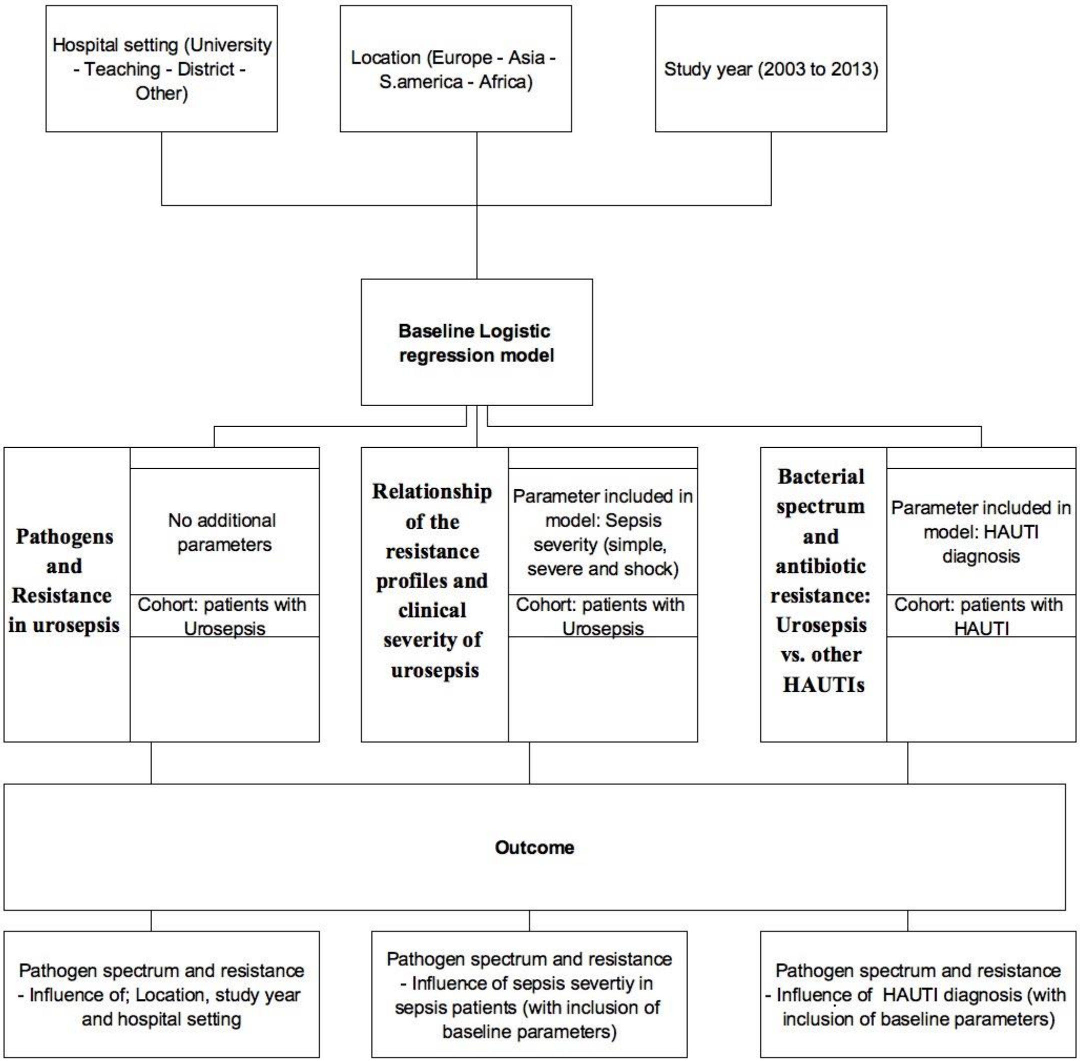

eFigure 4, Overall frequency of pathogens in consecutive study years.

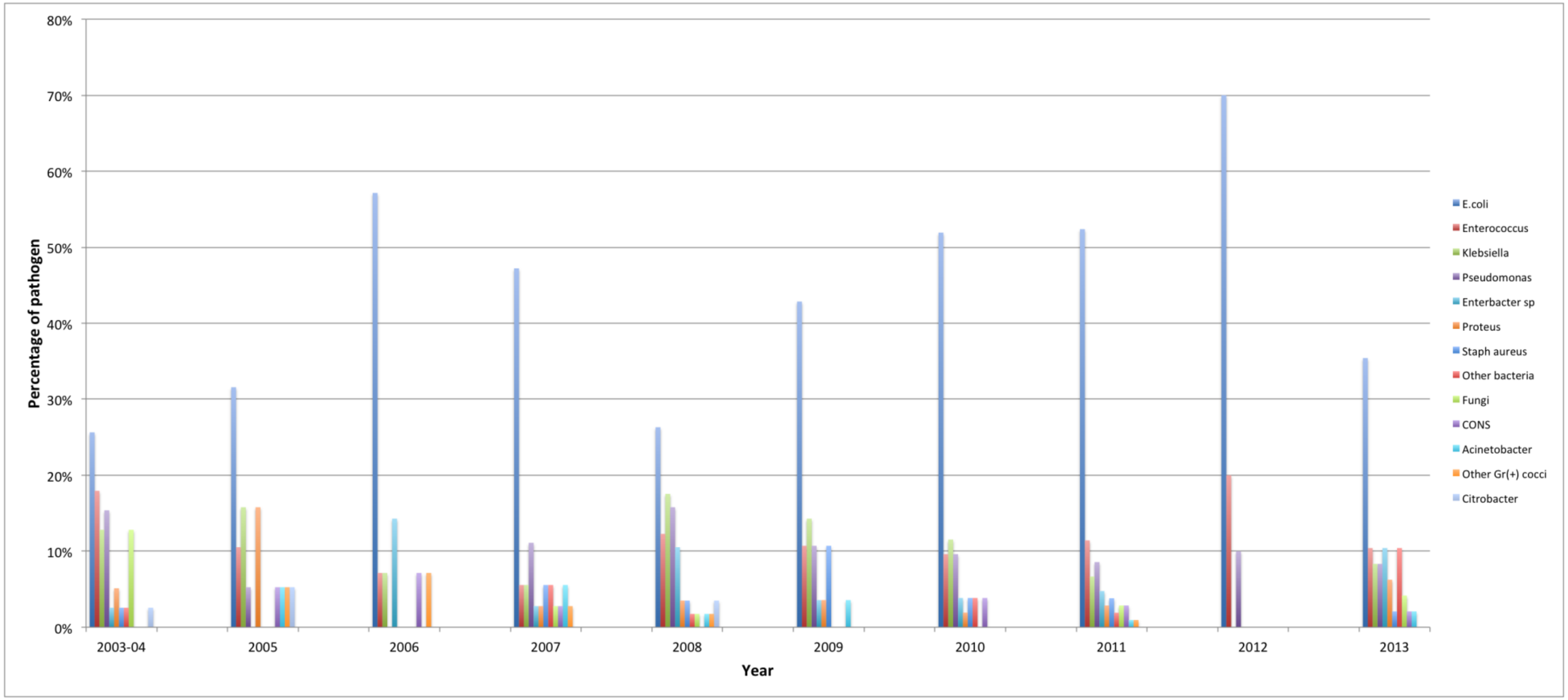

(CoNS=Coagulase negative staphylococcus)

Fluctuations in the pathogen spectrum frequency can be observed. However, there is no time trend associated with these fluctuations (multiple logistic regression analysis, Likelihood-ratio p value>0.05)

eFigure-5, Annual resistance rates of antibiotics in urosepsis. Fluctuations in resistance can be seen, however on the overall 11-year time period a significant change was not identified.

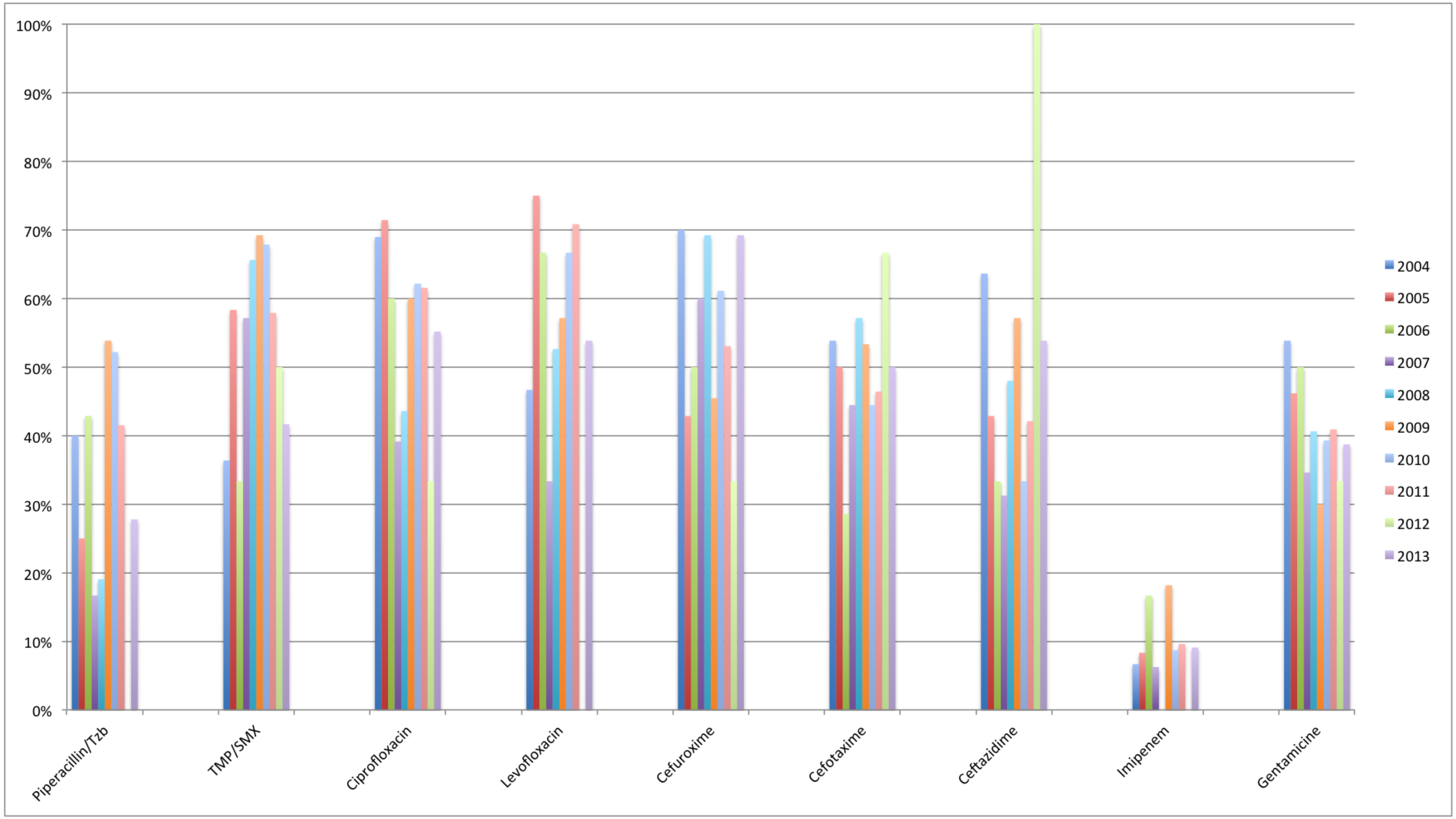

2. Tables

Table 1; Standards used for urine cultures susceptibility assessment in GPIU cohort

NCCLS	874	54,4%
DIN	171	10,6%
EUCAS	166	10,3%
Other	241	15%
Not provided	154	9,5%

Table 2; Pathogens and antimicrobials recorded in GPIU study forms

Pathogens	Antimicrobials
E. coli	Aminopenicillin (Ampicillin or Amoxicillin) in combination with a β-lactamase inhibitor (BLI; Sulbactam or Clavulanic acid)
Klebsiella spp.	Piperacillin in combination with Tazobactam
S. aureus	Trimethoprim in combination with Sulfamethoxazole
Proteus spp.	Ciprofloxacin
Morganella spp.	Cefuroxime
Enterobacter spp.	Cefotaxime
Enterococcus spp.	Ceftazidime
P.aeruginosa	Imipenem
CoNS	Gentamicin
Citrobacter spp.	
Acinetobacter spp.	
Other Gram-(+) cocci	
Other bacteria	
Candida albicans	
Other Fungi	

3. Classification of UTIs used in the GPIU Study

3.1. Introduction

Traditionally, UTIs are classified based on clinical symptoms, laboratory data, and microbiological findings. Practically, UTIs have been divided in uncomplicated and complicated UTIs, and sepsis. It is important to underline that the following proposed classification is still not validated or recognised. It is a working instrument.

A critical review of present classifications was undertaken for the EAU/ICUD Urogenital Infections initiative (1) in Appendix 16.1. The overall aim is to provide the clinician and researcher with a standardised tool and nomenclature for UTI. The present guidelines give a short summary of a tentative improved system of classification of UTI based on:

- anatomical level of infection;

- grade of severity of infection;

- underlying risk factors;

- microbiological findings.

The symptoms, signs and laboratory finding focus on the anatomical level and the degree of severity of the infection. The risk factor analysis contributes to define any additional therapeutic measure required (i.e. drainage).

3.2. Level of infection

The symptoms, as presented in the Appendix 16.1, focus on the level of infection, defined as:

- urethritis (UR);

- cystitis (CY);

- pyelonephritis (PN);

- sepsis (US).

Urethritis being poorly understood is for the time being not included in the algorithm and treatment strategy of pure UTI. The male genital infections prostatitis and epididymitis are also not included.

Asymptomatic bacteriuria (ABU) needs to be considered a special entity because it can have its source in both the lower and upper urinary tracts, and requires no treatment unless the patient is subjected to urological surgery.

3.3. Grade of severity

The grade of severity is set on a scale of 1-6 that is related to the risk of fatal outcome (Figure 1)

Figure 1: Traditional and improved classification of UTI as proposed by the EAU European Section of Infection in Urology (ESIU) (1)

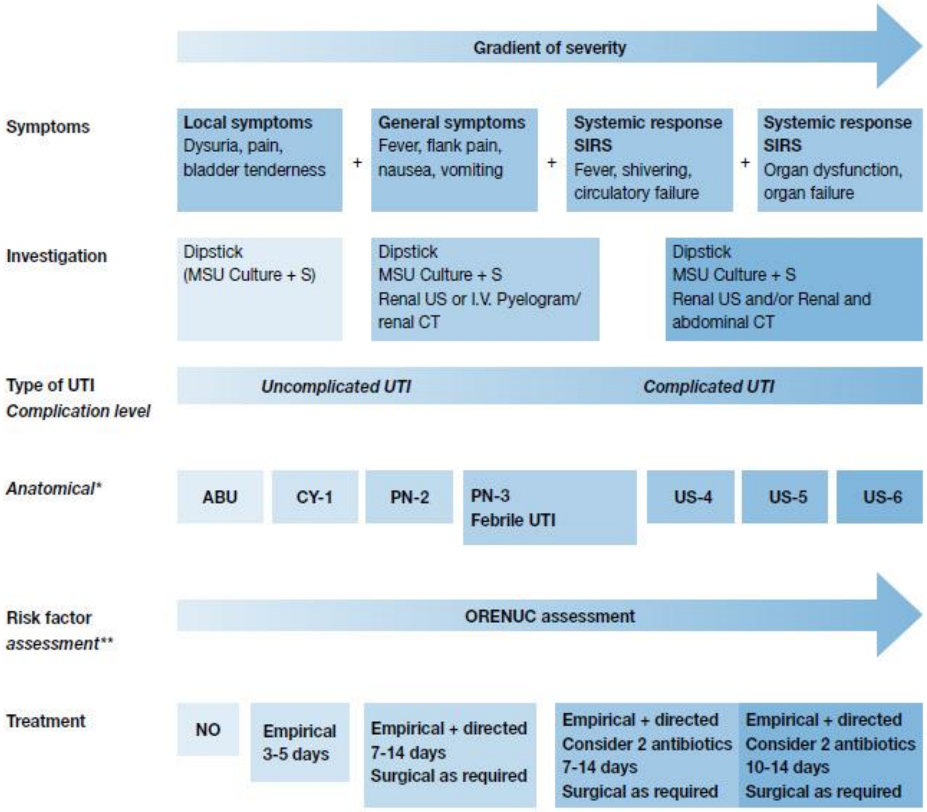

* see Figure 2

** see Table 1

Table 1: Host risk factors in UTI

Type	Category of risk factor	Examples of risk factors
O	No known/associated RF	- Healthy premenopausal women
R	RF of recurrent UTI, but no risk of severe outcome	- Sexual behaviour and contraceptive devices<br><br>- Hormonal deficiency in post menopause<br><br>- Secretory type of certain blood groups<br><br>- Controlled diabetes mellitus
E	Extra-urogenital RF, with risk or more severe outcome	- Pregnancy<br><br>- Male gender<br><br>- Badly controlled diabetes mellitus<br><br>- Relevant immunosuppression*<br><br>- Connective tissue diseases*<br><br>- Prematurity, new-born
N	Nephropathic disease, with risk of more severe outcome	- Relevant renal insufficiency*<br><br>- Polycystic nephropathy
U	Urological RF, with risk or more severe outcome, which can be resolved during therapy	- Ureteral obstruction (i.e. stone, stricture)<br><br>- Transient short-term urinary tract catheter<br><br>- Asymptomatic Bacteriuria**<br><br>- Controlled neurogenic bladder dysfunction<br><br>- Urological surgery
C	Permanent urinary Catheter and non resolvable urological RF, with risk of more severe outcome	- Long-term urinary tract catheter treatment<br><br>- Non resolvable urinary obstruction<br><br>- Badly controlled neurogenic bladder

RF = Risk Factor; * = not well defined; ** = usually in combination with other RF (i.e. pregnancy, urological internvention).

3.4. Pathogens

Urine culture will usually identify the causative pathogen (≥ 10^4 cfu/mL) and its susceptibility pattern. Both characteristics can be introduced in the final classification of the clinical stage of infection. The degree of susceptibility is defined as grade a (susceptible) to c (resistant).

3.5. UTI Classification

Figure 2 shows a summary of the additive parameters that make up an individual class of UTI.

Figure 2: Additive parameters of UTI classification and severity assessment

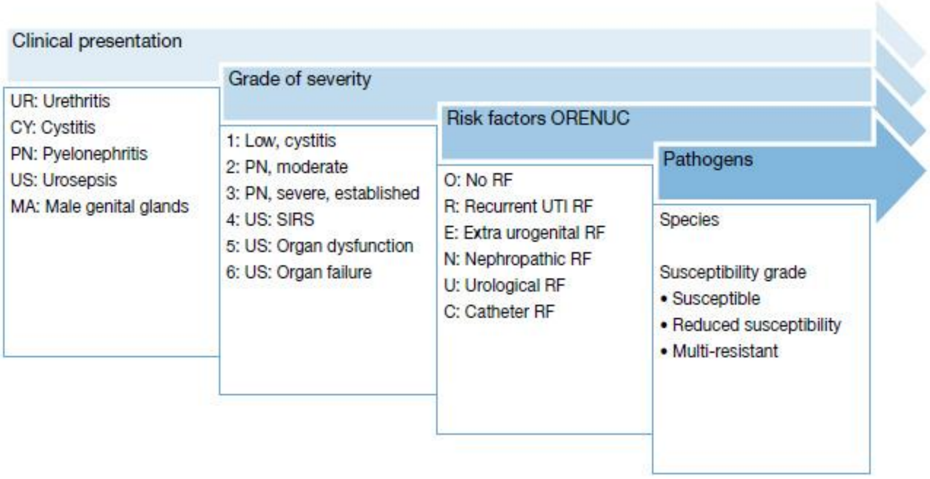

By cumulating the different parameters, a UTI can be classified as follows (1):

- CY-1R: E. coli (a): simple cystitis but recurrent with susceptibility to standard antibiotics.

- PN-3U: K pneumonia (b): severe pyelonephritis (with high fever and vomiting), with underlying urological disease (e.g. stones or obstruction) due to Klebsiella sp., with a moderate antibiotic resistance profile.

- US-5C: Enterococcus sp (a): severe urosepsis with an antibiotic-sensitive Enterococcus sp. in a patient with an indwelling catheter.

4. CDC Definitions Used in the GPIU study for diagnosis of HAUTI,[1,2,3]

Urinary Tract Infection

Urinary tract infection includes symptomatic urinary tract infection, asymptomatic bacteriuria, and other infections of the urinary tract.

Symptomatic urinary tract infection must meet one of the following criteria:

1. One of the following: fever (>38°C), urgency, frequency, dysuria, or suprapubic tenderness AND a urine culture* of >= 10^5 colonies/ml urine with no more than two species of organisms
2. Two of the following: fever (>38°C), urgency, frequency, dysuria, or suprapubic tenderness AND any of the following:
   i. Dipstick test positive for leukocyte esterase and/or nitrate
   ii. Pyuria (>=10 white blood cells (WBC)/ml or >=3 WBC/high-power field of unspun urine)
   iii. Organisms seen on Gram stain of unspun urine
   iv. Two urine cultures with repeated isolation of the same uropathogen[+] with 10^2 colonies/ml urine in nonvoided specimens
   v. Urine culture with <=10^5 colonies/ml urine of single uropathogen in patient being treated with appropriate antimicrobial therapy
   vi. Physician's diagnosis
   vii. Physician institutes appropriate antimicrobial therapy
3. Patient <=12 months of age has one of the following: fever (>38°C), hypothermia (<37°C), apnea, bradycardia, dysuria, lethargy, or vomiting AND urine culture of 10^5 colonies/ml urine with no more than two species of organisms
4. Patient <=12 months of age has one of the following: fever (>38°C), hypothermia (>37°C), apnea, bradycardia, dysuria, lethargy, or vomiting AND any of the following:
   i. Dipstick test positive for leukocyte esterase and/or nitrate
   ii. Pyuria
   iii. Organisms seen on Gram stain of unspun urine
   iv. Two urine cultures with repeated isolation of same uropathogen with >=10^2 organisms/ml urine in nonvoided specimens
   v. Urine culture with <=10^5 colonies/ml urine of a single uropathogen in patient being treated with appropriate antimicrobial therapy
   vi. Physician's diagnosis
   vii. Physician institutes appropriate antimicrobial therapy

Asymptomatic bacteriuria must meet either of the following criteria:

1. An indwelling urinary catheter is present within 7 days before urine is cultured AND patient has no fever (>38°C), urgency, frequency, dysuria or suprapubic tenderness AND has urine culture of 105 organisms/ml urine with no more than two species of organisms.
2. No indwelling urinary catheter is present within 7 days before the first of two urine cultures with >=10^5 organisms/ml urine of the same organism with no more than two species of organisms, AND patient has no fever(>38°C), urgency, frequency, dysuria, or suprapubic tenderness.

Other infections of the urinary tract (kidney, ureter, bladder, urethra, or tissues surrounding the retroperitoneal or perinephric spaces) must meet one of the following criteria:

1. Organism isolated from culture of fluid (other than urine) or tissue from affected site

2. An abscess or other evidence of infection seen on direct examination, during surgery, or by histopathologic examination
3. Two of the following: fever(>38°C), localized pain, or tenderness at involved site AND any of the following:
    1. Purulent drainage from affected site
    2. Organism isolated from blood culture
    3. Radiographic evidence of infection*
    4. Physican's diagnosis
    5. Physican institutes appropriate antimicrobial therapy
4. Patient <=12 months of age has one of the followin: fever (>38°C), hypothermia (<37°C), apnea, bradycardia, lethargy, or vomiting AND any of the following:
    i. Purulent drainage from affected site
    ii. Organism isolated from blood culture
    iii. Radiographic evidence of infection
    iv. Physician's diagnosis
    v. Physician instituted appropriate therapy

***For urine specimens to be of value in determining whether a nosocomial infection exists, they must be obtained aseptically using an appropriate technique, such as clean catch collection, bladder catheterization, or suprapubic aspiration.**

+Gram-negative bacteria of Staphylococcus saprophyticus

5. Clinical Diagnostic Criteria of Urosepsis and Uroseptic Shock used in the GPIU study

Disorder	Definition
Urosepsis (simple)	Activation of a systemic inflammatory response syndrome (SIRS) due to a urinary tract infection (UTI). This systemic response is manifested by two or more of the following conditions:<br>Temperature > 38°C or < 36°C<br>Heart rate > 90 beats min<br>Respiratory rate > 20 breaths/min or PaCO2 < 32mmHg (< 4.3kPa)<br>WBC > 12,000 cells/mm^3 or < 4,000 cells/mm^3 or ≥ 10% immature (band) forms
Severe Urosepsis	Urosepsis associated with organ dysfunction, hypoperfusion or hypotension.<br>Hypoperfusion and perfusion abnormalities may include but are not limited to lactic acidosis, oliguria or an acute alteration of mental status
Uroseptic Shock	Urosepsis with hypotension despite adequate fluid resuscitation along with the presence of perfusion abnormalities that may include, but are not limited to lactic acidosis, oliguria, or an acute alteration in mental status.<br>Patients who are on inotropic or vasopressor agents may not be hypotensive at the time that perfusion abnormalities are measured

6. *References*

1. Bjerklund Johansen T E, Botto H, Cek M, Grabe M et al. Critical review of current definitions of urinary tract infections and proposal of an ESU/ESIU classification system. Internat J Antimicrob Agents 2011;38S:64-70.

2. Horan TC, Andrus M, Dudeck MA. *CDC/NHSN surveillance definition of health care-associated infection and criteria for specific types of infections in the acute care setting.* Am J Infect Control 2008, 36(5): 309-32.

3. adjusted to urology: Naber KG, Schaeffer AJ, Heyns CF, Matsumoto T, Shoskes DA, Bjerklund Johansen TE (eds): *Urogenital Infections. European Association of Urology - International Consultation on Urological Diseases*, 1st edition 2010, Arnhem, The Netherlands, ISBN:978-90-79754-41-0

4. GPIU Investigators

Christoph		Zorn	Konstanz	Germany
Muharrem	Murat	YILDIZ	ANKARA	Turkey
Oleg	Nickolay	Zuban	Saint-Petersburg	Russia
Konstantinos		Zougkas	Rhodes	Greece
Jose Renato	Ferreira	Zottich	Rio De Janeiro	Brazil
Srdjan	Dusan	Zivojinov	Novi Sad	Serbia and Montenegro
Andrea		Zitella	Turin	Italy
Reinhold	P	Zimmermann	Kufstein	Austria
Matthias		Zimmermann	Zurich	Switzerland
Nikolay	Viktorovich	Zhuravlev	Krasnoobsk	Russia
Lan	Ru	Zhu	Wuhan	China
Evisa		Zhapa	Tirana	Albania
Sde	De Cãƒâ¡Ssio	Zequi	Sao Paulo	Brazil
Bachar		Zelhof	Preston	United Kingdom
Luis	Rafael	Zegarra	Lima	Peru
Romuald		Zdrojowy	Warsaw	Poland
Sebastian		Zbrun	Bern	Switzerland
Andrey	Vladimirovich	Zaytsev	Moscow	Russia
Mihhail		Zarkovski	Tartu	Estonia
Gianpaolo		Zanetti	Milan	Italy
Morad	Abdallh	Zaki	Sohag	Egypt
K	Kuruvilla	Zachariah	Ashington	United Kingdom
Maher	Fawzi	Zabaneh	Amman	Jordan
Muhammad	Kabiru	Yusuf	Kano	Nigeria
Buhalov	Yuri	Yuri	Kyiv	Ukraine
Ilkan		Yuksel	Edirne	Turkey
Abbas		Yousefi Rad	Ankara	Turkey
Jeong	Jae	Young	Inchun	Korea, South
Ma		Yong	Shandong	China
Gul	Ruhsar	Yilmaz	Ankara	Turkey
Mustafa		Yildirim	Manisa	Turkey
Asif		Yildirim	Istanbul	Turkey
Wang		Yi	Shen Yang	China
Aydin		Yenilmez	Eskisehir	Turkey
Ozgur		Yaycioglu	Adana	Turkey
Ismet		Yavascaoglu	Bursa	Turkey
Stephen Shei- Dei		Yang	New Taipei City	Taiwan
Nguyen	Dinh	Xuong	Ho Chi Minh	Vietnam
Chao	Guan	Xu	Shiyan	Armenia
Yuan		Xiaoyi	Wuhan	China
Zhang		Xiangbo	Lanzhou	China

Fiona	Mei Wen	Wu	Singapore	Singapore
Eiling		Wu	Plymouth	United Kingdom
Eric	Roger	Wroclawski	Santo Andraja	Brazil
Slawomir	Pawel	Wozniak	London	United Kingdom
Ming Kui		Wong	W. P. Labuan	Malaysia
Gregory	Johann	Wirth		Switzerland
Joerg		Winkle	Aschaffenburg	Germany
Anika		Winkel	Recklinghausen	Germany
Florian	Thomas	Wimpissinger	Vienna	Austria
Simon		Williams	Derby	United Kingdom
Jan	Van Der	Wijk	STADSKANAAL	Netherlands
Thamara		Wijesuriya	Ragama	Sri Lanka
Jacqueline		Wicht	Brandenburg	Germany
Veronica		Weterings	Breda	Netherlands
Martin		Westenfelder	Krefeld	Germany
Peter		Weib	Siegen	Germany
Madhav	Harihar	Waze	Panjim	India
Graham	Michael	Watson	Near Polegate	United Kingdom
Yi		Wang	Shengyang	China
Wei		Wang	Beijing	China
Mikala		Wang	Aarhus N	Denmark
Alexander	Arkadievich	Walsky	Ninsk	Belarus
Raphaela	Maria	Waidelich	Munich	Germany
Florian	Martin Erich	Wagenlehner	Giessen	Germany
Koichiro		Wada	Okayama	Japan
Vinka		Vukotic	Belgrade	Serbia and Montenegro
Chuyen		Vu Le	Ho Chi Minh	Vietnam
Le	Nguyen	Vu	Hanoi	Vietnam
Jaroslav		Vseticka	Jablonec Nad Nisou	Czech Republic
Theodore		Voudoukis	Aeghion	Greece
Nikolay	Andreevich	Vorobyov	Saint-Petersburg	Russia
Martin		Vorauer	Baden	Austria
Martin		Vorauer	Baden	Austria
Friedrich	Paul	Von Toggenburg	St. Gallen	Switzerland
Bjoern	Georg	Volkmer	Ulm	Germany
Thomas	Daniel	Vogt	Ergolding	Germany
Martin		Vogelsang	Ulm	Germany
Yannis		Vlamopoulos	Lausanne	Switzerland
Starodoubtsevan	Nadia	Vladimirovna	Pervouralsk	Russia
Maltsev	Andrey	Vladimirovich	Donetsk	Ukraine
Luchitskiy		Vitaliy	Kiyv	Ukraine
Jaspal	S	Virdi	Harlow,Essex	United Kingdom
Alexey	Dmitrievich	Vinokurov	Saint-Petersburg	Russia
Nataliya	Andreevna	Vinarova	Moscow	Russia

Brigitta		Villumsen	Holstebro	Denmark
Artsem		Viliukha	Minsk	Belarus
Angelo M.		Viggiano	Leon	Spain
Nguyen	Phu	Viet	Hanoi	Vietnam
Alberto		Vianello	Perugia	Italy
Tibor		Veszpremi		Hungary
Mr Prateek		Verma	Eastbourne	United Kingdom
Lisandro	Ignacio	Veliz	Buenos Aires	Argentina
Valia	Valentinova	Veleva	Sofia	Bulgaria
Muthu	Veeramani	Veeramani	Nadiad	India
Zoltan		Varga	Sigmaringen	Germany
Gabriel		Varga	Brno	Czech Republic
Marco		Varaldo	Genova	Italy
Paul		Van Wijk	Amsterdam	Netherlands
Jan	Paul	Van Haverbeke	Tielt	Belgium
Nathalie		Van Der Mee-Marquet	Tours	France
Michael	Rogier	Van Balken	Arnhem	Netherlands
Mstislav	Morozov	Valentinovich	Smolensk	Russia
Haluk		Vahaboglu	Izmit	Turkey
Markku	H	Vaarala	OYS	Finland
Nuray		Uzun	Istanbul	Turkey
Akylbek	Ch.	Usupbaev	Bishkek	Kyrgyzstan
Enzo		Usai	Cagliari	Italy
Juan		Uria	Vic	Spain
Suleyman	Erdinc	Unluer	Istanbul	Turkey
Dogan		Unal	Ankara	Turkey
Soner		Ulusoy	Istanbul	Turkey
Tomohiro		Ueda	Kyoto	Japan
Md Mohsin		Uddin	Dhaka	Bangladesh
Vasilios		Tzortzis	Larissa	Greece
Tahsin		Turunc	ADANA	Turkey
Bruce		Turner	London	United Kingdom
Levent	Niyazi	Turkeri	Istanbul	Turkey
Polat		Turker	Istanbul	Turkey
Cane	Dz	Tulic	Belgrade	Serbia and Montenegro
Muzaffar		Tukhtamishev	Tashkent	Uzbekistan
Andrea		Tubaro	Rome	Italy
Anton		Tsukanov	Omsk	Russia
Nadiya	Stepanivna	Tselyukh	Lviv	Ukraine
Petros		Tsafrakidis	Thessaloniki	Greece
Urszula	Grazyna	Trykozko	Warsaw	Poland
Andrej		Trusila	Minsk	Belarus
Tron		Tronsen	Levanger	Norway

Alberto		Trinchieri	Lecco	Italy
Yulia	Pavel	Trifonova	Kiev	Ukraine
Khac Linh		Tran Ngoc	Ho Chi Minh City	Vietnam
Nguyen	Van	Tran	Cantho	Vietnam
Svetia	Jordanov	Toujarov	Sofia	Bulgaria
Csaba		Toth	Debrecen	Hungary
Fettah		Tosun	Istanbul	Turkey
Gina	Marcela	Torres Zambrano	Madrid	Spain
Joãƒâ£O	Pimentel	Torres	Braga	Portugal
Jesus	Ignacio	Tornero	Murcia	Spain
Mohamed	Kotb	Tolba	Cairo	Egypt
Gokhan		Toktas	Istanbul	Turkey
Piero	Giovanni	Tognoni	Bogliasco-Genoa	Italy
Ilker		Tinay	Siirt	Turkey
Marco		Tiberi	Trento	Italy
Yahya	Tfeil	Tfeil	Nouakchott	Mauritania
Petr		Tesar	Praha 4	Czech Republic
Peter		Tenke	Budapest	Hungary
Chu	Leong	Teh	Georgetown	Malaysia
Alaa	Abdallah	Tealab	Madinah Menwarra	Saudi Arabia
Maria Fe	Raymundo	Tayzon	Pasig City	Philippines
Meltem		Tasbakan	Izmir	Turkey
Msasanobu		Tanimura	Kochi-City	Japan
Neelam		Taneja	Chandigarh	India
Zafer		Tandogdu	Istanbul	Turkey
Kazushi		Tanaka	Kobe	Japan
Toomas		Tamm	Tallinn	Estonia
Arjana		Tambic Andrasevic	Zagreb	Croatia
Perepanova	Sergeevna	Tamara	Moscow	Russia
Nador		Tamas	Budapest	Hungary
Kaisar	Ali	Talukder	Dhaka	Bangladesh
Satoshi		Takahashi	Sapporo	Japan
Koichi		Takahashi	Onga Gun	Japan
Hamid	Reza	Tajari	Gorgan	Iran
Mehrdad		Tahami	Urmia	Iran
Ali		Taghizade Afshari	Urmia	Iran
Rahim	Razavi	Taghavi	Mashhad	Iran
Ferenc	-	Torzosk	G.	Hungary
Ervin		Szentgyorgyi	Vac	Hungary
Tamas		Szeberenyi	Gyula	Hungary
Andras		Szathmari	Miskolc	Hungary
Istvan		Szalay	Szeged	Hungary
Zoltan		Szabo	Kecskemet	Hungary
Clare		Sweeney	Aberdeen	United Kingdom

Joumana	Issa	Sunna	Amman	Jordan
Paul Anthony	Lugue	Sunga	Quezon City	Philippines
Fredrik		Sunden	Helsingborg	Sweden
Vesna	D	Suljagıc	Belgrade	Serbia and Montenegro
Branislav	Lazar	SUBOTIC	Brig	Switzerland
Nazareno		Suardi	Milan	Italy
Sergiy	Petrovych	Styopushkin	Dnipropetrovsk	Ukraine
Viveka	Diana	Strock	Goteborg	Sweden
Miroslav	Milorad	Stojadinovic	Kragujevac	Serbia and Montenegro
Ingunn		Steingrimsdottir	Reykjavãƒâk	Iceland
Dan		Stefanescu	Bistrita	Romania
Sotir		Stavridis	Skopje	Macedonia, The Former Yugoslav Republic of
Vladimir		Startsev	St.-Petersburg	Russia
Lesnikov	Vladimir	Stanislavovich	Donetsk	Ukraine
Maria	Ines	Staneloni	Buenos Aires	Argentina
Roman		Stanek	Opava	Czech Republic
Konstantinos		Stamatiou	Pireas	Greece
Pia		Stallenberg	Amsterdam	Netherlands
Annabelle		Stainier	Yvoir	Belgium
Christopher		Springer	Vienna	Austria
Annabel		Spek	Meiningen	Germany
Bjãƒâ¶Rn		Specht	Stuttgart	Germany
Hajrudin		Spahovic	Sarajevo	Bosnia and Herzegovina
Philipp	Julian	Spachmann	Regensburg	Germany
Petros		Sountoulides	Veria	Greece
Michelangelo		Sorrentino	Napoli	Italy
Jãƒâ¶Rg		Sommer	Lohne	Germany
Gyorgy	-	Solt	Harlow	United Kingdom
Thong		Sok Hean	Phnom Penh	Cambodia
Maja		Sofronievska Glavinov	Skopje	Macedonia, The Former Yugoslav Republic of
Mustafa		Sofikerim	Kayseri	Turkey
Doddy	M.	Soebadi	Surabaya	Indonesia
Chavdar	Krumov	Slavov	Sofia	Bulgaria
Aliaksandr		Sivets	Minsk	Belarus
Liubov	Alexandrovna	Sinyakova	Moskow	Russia
Rachhpal	S	Singh	Amritsar	India
Avinash	Kumar	Singh	Sofia	Bulgaria
Dorin		Singeorzan	Miercurea Ciuc	Romania
Orhun		Sinanoglu	Istanbul	Turkey

Battisti		Simon	La Roche Sur Yon Cedex 9	France
Adrian	Diego	Simoes	Canterbury	United Kingdom
Ricardo	Pereira E	Silva	Lisboa	Portugal
Yoram	Itchak	Siegel	Zerifin	Israel
Marcin		Sieczkowski	Gdansk	Poland
Maksim		Sidorenko	Kiev	Ukraine
Babu Vijayakumar		Sidharaju	Salem	India
Alexander	Vladislavovich	Shuliak	Kiev	Ukraine
Mitra		Shodjai-Baghini	Vienna	Austria
Takehiko		Sho	Kitakyushu	Japan
Dimiter	Grigorov	Shishkov	Plovdiv	Bulgaria
Bongsuk		Shim	Seoul	Korea, South
Katsumi		Shigemura	Kobe	Japan
Alexey	Alexandrovich	Shevyrin	Ivanovo	Russia
Amir	Hossein	Sharafi	Urumia	Iran
Sergey	Nikolaevich	Shamrayev	Donetsk	Ukraine
Hassan	Sayed	Shaker	Cairo	Egypt
Mimoza	Ekrem	Shaipi	Presevo	Serbia and Montenegro
Syed	Ali	Shahzad	SLOUGH	United Kingdom
Garnik		Shahbazyan	Yerevan	Armenia
Nexhat	Emin	Shabani	Gjilan/Kosovo	Albania
Stavros	Fotios	Sfoungaristos	Karpathos	Greece
Musteba		Sevil	Afyon	Turkey
Joanne	Slyth	Serrano Uribe	Alcala De Henares	Spain
Alexander	Alexandrovitch	Seriogin	Moscow	Russia
Maroun		SERHAL	Beirut	Lebanon
Anup		Sengupta	Bury St. Edmunds	United Kingdom
Yavor	Petrov	Semerdzhiev	Sofia	Bulgaria
Mehmet	Bulent	Semerci	Izmir	Turkey
Oscar		Selvaggio	Matera	Italy
Ilker		Seckiner	Gaziantep	Turkey
Dr..Joseph	Philipraj	Sebastian	Gangtok	India
Alessandro		Sciarra	Rome	Italy
Bernhard	Michael	Schwindl	Weiden	Germany
Martin		Schoenthaler	Freiburg	Germany
Stefan		Schoeler	Mãƒâ¼Nchen	Germany
Ludvik		Schnitzer	Praha 4	Czech Republic
Marcus		Schenck	Essen	Germany
Markus		Schãƒffer	Coburg	Germany
Markus		Schãƒâ¤Fer	Coburg	Germany
Sabrina		Schaeffer	Garmisch-Partenkirchen	Germany
Markus		Schãƒâ¶Nberger	K.	Germany
Anthony	J	Schaeffer	Chicago	United States

Roberto	Mario	Scarpa	Orbassano	Italy
Ariel	Gustavo	Scafuri	Fortaleza, Ceara	Brazil
Mark	Fraser	Saxby	Stoke-On-Trent	United Kingdom
Savvas	Tryfon	Savvaidis	Argos	Greece
Orlin		Savov	Nuremberg	Germany
Muppidi		Satyavani	Bandar Seri Bagawan	Brunei
Karen		Sarkisyan	Saint-Petersburg	Russia
Mehrdad		Sareh	Bucharest	Romania
Rosanna	Tubo	Santillan	Makati City	Philippines
Sandro	Danilo	Sandri	Magenta	Italy
Gecs		Sandor	Veszprãƒâ©M	Hungary
Susanna	Malin	Sandberg	Karlstad	Sweden
Murat		Samli	Afyon	Turkey
Hassan	Mikhael	Saloum	Riyadh	Saudi Arabia
Ahmed		Salman	Lahore	Pakistan
Sataa		Sallami	Tunis	Tunisia
Hosni	Khairy	Salem	Cairo	Egypt
Mohammad		Salehi	Rasht	Iran
Leonardo	Mose	Salame	Cefalu (Palermo)	Italy
Morshed	Ali	Salah	Al Wakra	Qatar
Vasileios	I	Sakalis	Thessaloniki	Greece
Radhia		Saidi	Monastir	Tunisia
Kapil		Sahnan	Gloucestershire	United Kingdom
Tayfun	-	Sahinkanat	Kahramanmaras	Turkey
Hayrettin		Şahin	Diyarbakir	Turkey
Erkin		Saglam	Istanbul	Turkey
Ahmed	S	Safwat	Assiut	Egypt
Refat	Abdelsamie	Sadeq	Zagazig	Egypt
Nourkhoda		Sadeghifard	Ilam	Iran
Emilio		Sacco	Roma	Italy
Brookman-Amissah		Sabine	Weiden	Germany
Sherif	Helmy	Saafan	Cairo	Egypt
M	M	S	Fayoum	Egypt
Emile		Rwamasirabo	Kigali	Rwanda
Andras		Rusz	Giessen	Germany
Annebeth		Ruiter	Nieuwegein	Netherlands
Robert	Raimund	Rudolph	Kirchheim	Germany
Levente		Rosztãƒâ³Czy	Baja	Hungary
Ãƒâ Gnes		Rosecker	Szeged	Hungary
Basilisk,	E	Rompis	Thessalonians	Greece
Imre		Romics	Budapest	Hungary
Alfonso	J.	Rodriguez-Morales	Trujillo	Venezuela
Viviana	M.	Rodriguez	Buenos Aires	Argentina
Cruz	N.	Rodriguez	Caracas	Venezuela
Raul	Nunes	Rodrigues	Angra Do	Portugal

			Heroismo	
Rafael		Rodrigues-Patraja	Madrid	Spain
Grãƒâ©Goire		Robert	Bordeaux	France
Torsten		Rith	Erlangen	Germany
Slobodan	Petar	Ristovski	Skopje	Macedonia, The Former Yugoslav Republic of
Pedro	M.	Rifakis	Caracas	Venezuela
Mehmet	Murat	Rifaioglu	Istanbul	Turkey
Hanna	Juliane	Richter	Garmisch-Partenkirchen	Germany
Carlos		Ribeiro Oliveira	Braga	Portugal
Theo	M	Reijke De	Amsterdam	Netherlands
Imre		Regos	Vac	Hungary
Harold	M	Red	Libertador San Martin	Argentina
Nouri		Rebai	Paris	France
Couti		Razvan	Cluj-Napoca	Romania
Dr	Syed Johar	Raza	Karachi	Pakistan
Raul		Raz	Afula	Israel
Mark		Rauthmann	Sigmaringen	Germany
Erika		Rauth	Pecs	Hungary
Deepak	Babu	Rauniyar	Dhulikhel	Nepal
Aso	Omer	Rashed	Sulaimani	Iraq
P.N.		Rao	Manchester	United Kingdom
Muhammad	Qamar Sarwar	Rana	Birmingham	United Kingdom
Anders		Ramsing	Vãƒâ¤Rnamo	Sweden
Rodrigo	Nuno Brito	Ramos	Lisboa	Portugal
Duje		Rako	Zagreb	Croatia
Lul		Raka	Prishtina	World
Hary Raj		Raja	Blackburn	United Kingdom
Syed	Imtiaz	Rahman	Newport	United Kingdom
Aino		Rãƒâµãƒâµm	Tallinn	Estonia
Muhammad		Rafique	MULTAN	Pakistan
Lili		Radulovic	Belgrade	Serbia and Montenegro
Sabri		Rachid	Casablanca	Morocco
Bryan	Ingemar	Qvick	Garmisch Partenkirchen	Germany
Ana Isabel	Linares	Quevedo	Madrid	Spain
Tahir	Uddin	Qazi	Kohat	Pacific Ocean
Wasim		Qasim	Dalian	China
Ralf		Pychynski	Bad Segeberg	Germany
Armin		Pycha	Bolzano	Italy
Praveen		Pushkar	New Delhi	India
Basuki	B	Purnomo	Malang	Indonesia

Tiziana		Puglisi	Ispica	Italy
Ignacio		Puche-Sanz	Granada	Spain
Petr		Prosvic	Nachod	Czech Republic
Jose-Vicente	Tablante	Prodigalidad	Quezon City	Philippines
Jose	Nestor	Procuna Hernandez	Mexico D.F.	Mexico
Domenico		Prezioso	Naples	Italy
Juan	Manuel	Poyato	Huelva	Spain
Mariela	Rosa	Pow-Sang	Lima	Peru
Tobias	Samuel	Pottek	Wedel	Germany
Martina		Porsch	Turnov	Czech Republic
Daniele		Porru	Pavia	Italy
Daniel	Hodade	Porav	Cluj-Napoca	Romania
Roberto		Ponchietti	Siena	Italy
Giorgio		Pomara	Pisa	Italy
Konstantinos		Polyzois	Manchester	United Kingdom
Hasan	S.	Pliev	Domodedovo	Russia
Alexei	Yurievich	Plekhanov	Saint-Petersburg	Russia
Pawel		Plaza	Lublin	Poland
Guido		Platz	Ruesselsheim	Germany
Mehmet	Mesut	Piskin	Konya	Turkey
Germar		Pinggera	Innsbruck	Austria
Mohan		Pillai	Blackburn	United Kingdom
Dirk		Piehler	Greifswald	Germany
Stefano		Picozzi	San Donato Milanese	Italy
David		Piccolotti	Lagosanto (FE)	Italy
Juan	Jose	Picazo	Madrid	Spain
Catherine	Elizabeth	Philps Pereira	Oslo	Norway
Joshua	Tarun	Phillips	Birmingham	United Kingdom
Yuriy	Yuryevich	Petrovskiy	Odessa	Ukraine
Georgios	Loizos	Petrikkos	Athens	Greece
John		Peters	London	United Kingdom
Melanie		Peter	Luebeck	Germany
Tunãƒâ«	P	Pervorfi	Prishtina	Serbia and Montenegro
Giacomo		Perugia	Rome	Italy
Dominik	Georg	Pernkopf	Vienna	Austria
Tamara	Sergeevna	Perepanova	Moscow	Russia
Miguel	Angel	Peredo	Mexico	Mexico
Dhelma	Isabel	Pellãƒân	Caracas	Venezuela
Miguel	Angel	Pedrola	Venado Tuerto	Argentina
Rizky		Paukstadt	Hof	Germany
Antonio	L	Pastore	Terracina (LT)	Italy
Sergii	Petrovich	Pasiechnikov	Kiev	Ukraine
Jai	Pal	Paryani	Abu Dhabi	United Arab Emirates
Pawel		Parniewski	Lodz	Poland

Shin	Jae	Park	Daegu	Korea, South
Seung Chol		Park	Iksan	Korea, South
Christos		Papandreou	Arta	Greece
Anestis	G.	Papadopoulos	Ptolemayda	Greece
Vlad		Pantea	Gelsenkirchen	Germany
Ioannis		Panopoulos	Athens	Greece
Manoj	Kumar	Panigrahi	Berhampur	India
Shiv	Kumar	Pandian	Colchester	United Kingdom
Sanjay		Pandey	Mumbai	India
Juan		Palou	Barcelona	Spain
Ivan	S.	Palagin	Smolensk	Russia
Andras		Pãƒâ¡Czelt	Budapest	Hungary
Hakan		Ozveri	Istanbul	Turkey
Ahmet		Ozturk	Konya	Turkey
Firas		Ozoe	Chambery	France
Hakan		Ozkardes	Ankara	Turkey
Tayyar	Alp	OZKAN	ISTANBUL &Kocaeli	Turkey
Ilhan		Ozgunes	Eskisehir	Turkey
Muhammet	Fuat	Özcan	Ankara	Turkey
Alexander	Georgiev	Otzetov	Sofia	Bulgaria
Michael		Osei-Tether	Kumasi	Ghana
Alva	Damaris	Ortiz	La Libertad	El Salvador
Mazhar		Ortaãƒâ§	Istanbul	Turkey
Samir	Shaaban	Orabi	Alexandria	Egypt
Chong Chien		Ooi	Selangor	Malaysia
Rahmi		Onur	Elazig	Turkey
Eng	Kwee	Ong	Barnstaple	United Kingdom
Daniel		Oliveira-Reis	Porto	Portugal
Kagan	Felixovich	Oleg	St.Persburg	Russia
Peter	Jochen	Olbert	Marburg	Germany
Taylan		Oksay	Isparta	Turkey
Athanassios		Oeconomou	Larissa	Greece
Yusuf	Vehbi	Ocak	Sanliurfa	Turkey
Gaga		Nutsubidze	Kutaisi	Georgia
Byron	F	Nunez Freile	Quito	Ecuador
Dmitry		Nitkin	Minsk	Belarus
Abolghasem		Nikfallah	Tehran	Iran
Heinz	Gerardo	Nicolai	Santiago	Chile
Dong	Le	Nguyen	Ho Chi Minh City	Vietnam
Roberto	Rossi	Neto	Essen	Germany
Elie	Ghannam	Nemr	Beirut	Lebanon
Hemant	Bhanudas	Nemade	Basildon	United Kingdom
Aleksei		Nelovkov	Tallinn	Estonia
Dana	Gabriela	Negru	Arad	Romania
Oktay		Nazlı	Izmir	Turkey

Stephen		Nazareth	Makati City	Philippines
Saygin		Nayman Alpat	Eskisehir	Turkey
Ala Eddin	Daud	Natsheh	Jerusalem	Israel
Matteo		Napoli	Trapani	Italy
Lukacs		Nandor	Kecskemet	Hungary
Djordje	Petar	Nale	Belgrade	Seychelles
Wataru		Nakamura	Tokyo	Japan
Haitham	Saeed	Nakad	Alswaida	Syria
Ali		Naghoni	Tehran	Iran
Akaki	A.N.	Nadareishvili	Tbilisi	Georgia
Samer		Nabolsi	Tulle	France
Ben Sorba		Nabil	Sousse	Tunisia
Kurt	G	Naber	Straubing	Germany
Yong-Gil		Na	Daejeon	Korea, South
Dmytro	Mykhailovich	Mykhailov	Kyiv	Ukraine
Nazim	-	Mutlu	KOCAELI	Turkey
Necmettin	Aydin	Mungan	Zonguldak	Turkey
Mehmet	Ugur	Mungan	Izmir	Turkey
Ismaila	A	Mungadi	Sokoto	Nigeria
Bakhadirkhanov	Mukhamed Zarif	Mukhamed Kabirkhonovich	Tashkent	Uzbekistan
Volker		Mueller-Mattheis	Duesseldorf	Germany
Kien	Alfred	Mteta	Moshi	Tanzania
Noreddin	Hamza	Msessa	Tripoli	Libya
Vladimir		Mozetic	Rijeka	Croatia
Javad	Nafchi	Moussavi	Szolnok	Hungary
Youssef		Moussa	Aleppo	Syria
Seyed Habibollah		Mousavi-Bahar	Hamadan	Iran
Emad Eldin	Khalid	Mousa	Sohar	Oman
Renato	Lains	Mota	LISBOA	Portugal
Giuseppe		Mostaccio	ESTE (PD)	Italy
Mohammad	Kazem	Moslemi	Qom	Iran
Saskia	Carmen	Morgenstern	Frankfurt Am Main	Germany
Rayo		Morfin	Guadalajara	Mexico
Manuel	F	Montesino	Eneriz	Spain
Myron	Sidonio	Monteiro	Bangor	United Kingdom
Nurbek	Kytaibekovich	Monolov	Bishkek	Kyrgyzstan
Gabriella		Mombelli	Magenta	Italy
Mayad	Nouma	Moktash	Najran	Saudi Arabia
Gholamreza		Mokhtari	Rasht	Iran
Tatyana	Nikolaevna	Moiseenko	Novosibirsk	Russia
Radman	Abdullah	Mohammed	Hodaidah	Yemen
Moudouni	Said	Mohammed	Marrakech	Morocco
Aza	A	Mohammed	Northampton	United Kingdom
Ahmed	Hedeia	Mohamed	Hurghada	Egypt

Mohammadreza		Moein	Yazd	Iran
Muhammad		Moazzam	Derby	United Kingdom
Lampros	Petros	Mitrakas	Larissa	Greece
Warli	Syah	Mirsya	Medan	Indonesia
Chen		Ming	Nanjing	China
Seung Ki		Min	Seoul	Korea, South
Stephan	M	Miller	Gelsenkirchen	Germany
Jose	Ignacio	Militello	Salta	Argentina
Karl	Joensen	Mikines	Herlev	Denmark
Vartolomei	M	Mihai Dorin	Ludus	Romania
Ilir	Iljaz	Miftari	Pristina	Albania
Jean-Claude		Miermont	Nimes	France
Ramunas		Mickevicius	Kaunas	Lithuania
Uwe	HG	Michl	Hamburg	Germany
Bernardino		Miãƒâ±Ana Lopez	Murcia	Spain
Naoufel		MIAADI	Rennes	France
Christian		Meyer	Hamburg	Germany
Denis	Abel	Mesa Borroto	Moron. Ciego De Avila	Cuba
Oguz		Mertoglu	Izmir	Turkey
Frank		Mennigen	Rheine	Germany
Violeta		Menendez Lopez	Sant Pere De Ribes	Spain
Massimo		Meneguolo	Belluno	Italy
Gustavo	Adolfo	Mendez	Posadas, Misiones	Argentina
Badaruddin	A	Memon	Khairpur	Pakistan
Michael	Dimitrios	Melekos	Larissa	Greece
Andreas		Meiãƒâ¸Ner	Bonn	Germany
Jose		Medina-Polo	Madrid	Spain
Jan		Mecl	Liberec	Czech Republic
Javier		Mayor De Castro	Madrid	Spain
Alexander		Maykhir	Nizhny Novgorod	Russia
Ivan	Victorovich	Matveyeu	Vitebsk	Belarus
Deliu-Victor		Matei	Milan	Italy
Maria		Mastruzzo	Loma Hermosa. Pcia De Buenos Aires	Argentina
Massimo		Massarelli	Ivrea	Italy
Goran	S	Marusic	Novi Sad	Serbia and Montenegro
Dean		Markiã	Rijeka	Croatia
Chiara		Mariani	Pisa	Italy
Andreas	E.A.	Manseck	Ingolstadt	Germany
Aditya		Manjunath	Bristol	United Kingdom
Cristian	Nicolae	Manea	Cluj Napoca	Romania

Amar	Kumar	Manandhar	Varna	Bulgaria
Annika		Malmquist	Ystad	Sweden
Ercan		Malkoc	Istanbul	Turkey
Bartosz	Radoslaw	Malkiewicz	Wroclaw	Poland
Anton	I.	Maliavin	Ulyanovsk	Russia
Saidamin	Anvarovich	Makhsudov	Tashkent	Uzbekistan
Julia		Makarycheva	Samara	Russia
Michael		Maier	Rothenburg O. D. Tauber	Germany
Mahmoud	Farouk	Mahmoud Awad	Zagazig,Sharqia	Egypt
Benatta		Mahmoud	Oran	Algeria
Pattabiraman		Maheshkumar	Kings Lynn	United Kingdom
Ida	Soo-Fan	MAH	Hong Kong	Hong Kong
Vittorio-Gianpaolo		Magri-Perletti	Milano	Italy
Giuseppe		Magistro	Olching	Germany
Massimo	-	Maffezzini	Genova	Italy
Rafael	Velazquez	Macias	Mexico	Mexico
Ciaran	B	Lynch	Birmingham	United Kingdom
Oliver		Luzar	Bonn	Germany
Nicolae		LUPSASCA	Vitry Le Franãƒâ§Ois	France
Jens-Claudio		Lunz	Regensburg	Germany
Martin		Ludwig	Marburg	Germany
Qiao		Ludong	Beijing	China
Shing-Hwa		Lu	Taipei City	Taiwan
Kevin		Lu	Kaohsiung	Taiwan
Mikhail	*	Lozinskiy	Trnava	Slovakia
Andrea		Loreto	Roma	Italy
Miguel	Angel	Lopez-Aramburu	Miranda De Ebro (Burgos)	Spain
Andres		Lopez De Alda	Don Benito, Badajoz	Spain
Pedro		Lopez Cubillana	Murcia	Spain
Sofia	Pinheiro	Lopes	Loures	Portugal
Sudhir	Kumar	Lokwani	Bhopal,M.P.	India
Joyce		Loeffler	Nice	France
Branimir		Lodeta	Varazdin	Croatia
Michele		Lodde	Bolzano	Italy
Umberto		Locunto	Firenze	Italy
Chiwen		Lo	New Taipei City	Taiwan
Eva		Ljunggren	Lund	Sweden
Bosas		Liudas	Kaunas	Lithuania
Anna	AL	Liskova	Nitra	Slovakia
Gabriele		Lindenau	Vienna	Austria
Teng Lung		Lin	Taipei	Taiwan
Donghoon		Lim	Gwangju	Korea, South

Di	Pi	Li	Nasice	Croatia
Gabriel	Isaac	Levy Hara	Buenos Aires	Argentina
Fredrik		Levin	Stockholm	Sweden
Philippe		Lesprit	Crãƒâ©Teil	France
Peter	Hasan	Leonidovich	Moscow	Russia
Dr. Mohamed	Said	Leithy	Alexandria	Egypt
Jãƒâ¶Rg		Leifeld	Leer	Germany
Michael	Yu	Leh	Pasig	Philippines
Seung-Ju		Lee	Suwon	Korea, South
Sang Don		Lee	Yansan	Korea, South
Hyun-Rim		Lee	Buchon-Si	Korea, South
Elaine	Wei Yin	Lee	Harrogate, North Yorkshire	United Kingdom
Chang-Ho		Lee	Cheonan	Korea, South
Angus		Lecuona	Cape Town	South Africa
Aleksandar		Lazic	Belgrade	Serbia and Montenegro
Jan	Henrik	Laurick	Hagen	Germany
Stefano		Lauretti	Roma	Italy
Firstname	Middlename	Lastname	City	Country
Roland		Lang	Landshut	Germany
Daniel		Landau	Beer Sheva	Israel
Michael		Lamche	Vienna	Austria
Akos		Lakatos	Szentes	Hungary
Muhammad	Shahzad	Laghari	Scunthorpe	United Kingdom
Marãƒâa	Josãƒâ©	Lãƒâ³Pez Furst	Buenos Aires	Argentina
Vanda	Daniela	Lãƒâ³Pez	Caracas	Venezuela
Hanga		Laszlo Miklos	Papa	Hungary
Pasquale	Gianfranco	La Rosa	Giarre (CT)	Italy
Christian		La Hmann	Ulm	Germany
Espen		Kvan	Drammen	Norway
Marjan	Jovan	Kuzmanoski	Skopje	Macedonia, The Former Yugoslav Republic of
Dimitri		Kuzenko	Uelzen	Germany
Ainura	Zarylbekovna	Kutmanova	Bishkek	Kyrgyzstan
Fatih	Osman	Kurtulus	Istanbul	Turkey
Marcin		Kurant	Bydgoszcz	Poland
Shlomo	Mario	Kuntze	Richterswil	Switzerland
Hiromi		Kumon	Okayama	Japan
Ekaterina	V.	Kulchavenya	Novosibirsk	Russia
Alexander		Kugler	Marktredwitz	Germany
Shoji		Kudou	Chuo Yamanahsi	Japan
Aleksandra		Krznar	Celje	Slovenia
Alexander	Nikolaevich	Kruglov	Moskow	Russia
Fabian		Kronschnabl	Landshut	Germany

Christian		Kriegel	Leipzig	Germany
Benedikt		Kreiner	Berlin	Germany
W.		Kramer	Bad Soden	Germany
Nicole	Petra	Kraischits	Vienna	Austria
Margus		Krabi	Tallinn	Estonia
Tamas	Bognar	Kozma	Zalaegerszeg	Hungary
Zafer		Kozacioglu	Izmir	Turkey
Peter		Kovãcs	Eger	Hungary
Grigorios		Kousidis	ATHENS	Greece
Sotirios	Athanasios	Koukos	Ioannina	Greece
Rumen	Pachev	Kotsev	Pleven	Bulgaria
Serghiy	Mykolayovich	Kotsar	Mukachevo	Ukraine
Sergey	Vladislavovich	Kotov	Moscow	Russia
Shigeru		Kosugi	Tokyo	Japan
Alexander	Yrevich	KOROLEV	Saratov	Russia
Laszlo		Koranyi	Sopron	Hungary
Kãroly		Könvyes	Szombathely	Hungary
Angelis		Konstantinopoulos	Patras	Greece
Tome	Nik	Komani	Gjakova-Kosova	Serbia and Montenegro
Sanjay		Kolte	Nagpur	India
Ivan		Kolombo	Turnov	Czech Republic
Nikolay		Kolev	Pleven	Bulgaria
Dirk		Koglin	Ulm	Germany
Mikhail	Josefovich	Kogan	Rostov On Don	Russia
Lukas		Kocis	Poprad	Slovakia
Ramazan		Kocaaslan	Istanbul	Turkey
Roberto		Knez	Trieste	Italy
Jacques		Klein	Geneva	Switzerland
Hiroshi		Kiyota	Tokyo	Japan
Stiliana		Kiurkchieva	Sofia	Bulgaria
Attila		Kiss	Budapest	Hungary
Jacek		Kis	Krasnystaw	Poland
Sinan		Kirecci	Istanbul	Turkey
Pernille	Skjold	Kingo	Aarhus N	Denmark
Quinten	Michael	King	Palmerston North	New Zealand
Th		Kim	Seoul	Korea, South
Kiho		Kim	Gyeongju	Korea, South
Kang Sup		Kim	Seoul	Korea, South
Jung Hoon		Kim	Seoul	Korea, South
Jun-Mo		Kim	Bucheon	Korea, South
Jong Il		Kim	Busan	Korea, South
Hong Bin		Kim	Seongnam	Korea, South
Chul Sung		Kim	Korea	Korea, South
Mehmet		KILINC	Konya	Turkey
Margot	Martyna	Kieruj	Bergisch	Germany

			Gladbach	
Iradj		Khosropanah	Rasht	Iran
Dinyar		Khazaeli	Ahvaz	Iran
Mhd	Mouayed	Khawajki	Damascus	Syria
Priya		Khanna	London	United Kingdom
Taskeen	Ahmad	Khan	Peshawar, NWFP	Pakistan
Shahid	Aziz Anwer	Khan	Redhill	United Kingdom
Farrukh	Kamal	Khan	Canterbury	United Kingdom
Faisal	Rauf	Khan	Newcastle Upon Tyne	United Kingdom
Rinat		Khammatov	Togliatti	Russia
Ismail	Mohamed	KHALAF	Cairo	Egypt
Karthi		Keyan	Cochin	India
Mehmet	Selcuk	Keskin	Ankara	Turkey
Gabor		Kerenyi	Eger	Hungary
Eray		Kemahli	Istanbul	Turkey
Nuri		Keimeroã„Å¸Lu	Bilecik	Turkey
Bastian		Keck	Erlangen	Germany
Fahimeh	-	Kazemi Rashed	Tabriz	Iran
Cevdet		Kaya	Istanbul	Turkey
Albert		Kaufmann	Cologne	Germany
Matthias		Katzmann	Bad Nauheim	Germany
Adham	Mohamad	Kassem	Beiruth	Lebanon
Simon		Kasa	Majuro	Marshall Islands
Kamila		Karmasova	Brno	Czech Republic
Ferhi		Karim	Rennes	France
Roman	Titus	Karig	Neuss	Germany
Neven		Kapun	Zagreb	Croatia
Rajni		Kapoor	Lucknow	India
Mohammad	Anas	Kanout	Coburg	Germany
Jacob		Kaneti	Beer-Sheva	Israel
Ktari	Ktari	Kamel	Monastir	Tunisia
Ahmed	Ismail	Kamel	Giza	Egypt
Odunayo		Kalejaiye	Plymouth	United Kingdom
Panagiotis		Kalafatis	Rhodos	Greece
Bela		Koves	Budapest	Hungary
Hikmet		Kãƒâ¶Seoã„Å¸Lu	IZMIR	Turkey
Mustafa		Kadihasanoglu	Istanbul	Turkey
Alina	No Name	Kaczmarek	Jozefoslaw	Poland
Brahim	Brahim	Kacem	Mostaganem	Algeria
Jan		Kaan	Utrecht	Netherlands
Suleyman		Karaca	Malatya	Turkey
Rozalia		Juraszik	Budapest	Hungary
Seung Il		Jung	Hwasun	Korea, South
Peter		Jung	Ellwangen	Germany
Noor	Nabi	Junejo	Karachi	Pakistan

Robert		Juhasz	Berettyoujfalu	Hungary
Kondas		Jozsef	Budapest	Hungary
Teemu	Juhani	Joutsi	Pori	Finland
Rafaãƒâ«L	M. Alexandre	Joseph	Ieper	Belgium
Bruno	Guerra	Jorge Pereira	Covilhãƒâ£	Portugal
John	Jared	Johnson	Madrid	Spain
Antonio		Jimãƒâ©Nez-Pacheco	Granada	Spain
Ho	Jong	Jeon	Daegu	Korea, South
Susanne	Skou	Jensen	Aarhus N	Denmark
Ursula		Janzen	Hannover	Germany
Jiri		Janu	Nove Mesto Na Morave	Czech Republic
Vãƒâ¡Clav		Janda	Plzeã…Ë†	Czech Republic
Basharat	-	Jameel	Aberystwyth	United Kingdom
Lotte	Kaasgaard	Jakobsen	Aarhus	Denmark
Ramasamy		Jaganathan	Solihull	United Kingdom
Syed	Qasim	Jaffry	Galway	Ireland
Alessandro		Izzo	Napoli	Italy
Urii		Ivanov	Ekateringburg	Russia
George	Valerievich	Ivanov	Minsk	Belarus
Dmitri	D	Ivanov	Kiev	Ukraine
Untila	Victor	Iurie	Chisinev	Moldova
Rakasz		Istvan	Kaposvãƒâ¡R	Hungary
Vass		Istvãƒâ¡N	Miskolc	Hungary
Jamoliddin	Abdullajanov	Isroilovich	Namangan	Uzbekistan
Mohamed		Ismail	Bristol	United Kingdom
Noritaka		Ishito	Kurashiki	Japan
Satoshi		Ishihara	Minokamo, Gifu	Japan
Ekaterina	Martjushova	Irgens	Tromsãƒâ¸	Norway
Jacques		Irani	Poitiers	France
Beniamino		Iorio	Roma	Italy
Hisato		Inatomi	Fukutsu	Japan
Hodi		Imre	Szolnok	Hungary
Ben Salah		Imed	Bizerte	Tunisia
Anthony	Chika	Igbokwe	Lagos	Nigeria
Alaa	Ahmed	Hussein	Cairo	Egypt
Akhtar Hussain		Hussain	Slough	United Kingdom
Emre		Huri	Ankara	Turkey
Tobias		Huebschle	Freiburg	Germany
Xiaoming		Huang	Shenyang	China
Andras		Hoznek	Creteil	France
Mustafa Burak		Hoşcan	Alanya-Antalya	Turkey
Titia	EM	Hopmans	Utrecht	Netherlands
Adel	Abdulkader	Hommos	Maadi, Cairo	Egypt

Petr		Holy	Praha	Czech Republic
Sten		Holmãng	Goteborg	Sweden
Endre		Holman	Kiskunhalas	Hungary
Reinhard	Hans Hermann	Hofmann	Wolfsburg	Germany
Harun		Hodzic	Zenica	Bosnia and Herzegovina
Andreas		Hinkel	Herne	Germany
Alexander	Ivanov	Hinev	Varna	Bulgaria
Mustafa		Hilmy	Stirling	United Kingdom
Vital		Hevia	Madrid	Spain
Jorge		Hernandez	Panama	Panama
Javier	Alejandro	Hernandez	Barquisimeto	Venezuela
David		Hernanadez	Santa Cruz De Tenerife	Spain
Jiri		Heracek	Prague	Czech Republic
Derek	B	Hennessey	Derry	United Kingdom
Flavio	L	Heldwein	Florianãƒâ³Polis	Brazil
Burkhard	Johannes	Held	Berlin	Germany
Mohamed Hani	Abdulwahab	Helal	Jeddah	Saudi Arabia
Ortwin		Heiãƒå¸Ler	Gansersdorf	Austria
Hiroshi		Hayami	Kagoshima	Japan
Simon	John	Hawkyard	Scarborough	United Kingdom
Botan		Hawas	Vasteras	Sweden
Gergely		Hausmann	Budapest	Hungary
Dinko		Hauptman	Zagreb	Croatia
Konstantinos		Hatzimouratidis	Pefka Thessaloniki	Greece
Ismail	M	Hassan	Dubai	United Arab Emirates
Hashim		Hashim	London	United Kingdom
Waleed	Ali	Hasan	Manama	Bahrain
Sergio	Pedro	Haro	Tromsoe	Norway
Ivan	Iovev	Handjiev	Varna	Bulgaria
Vishwanath	S	Hanchanale	York	United Kingdom
Chang	Hee	Han	Uijeongbu	Korea, South
Arman	H.	Hambaryan	Echmiadzin	Armenia
Ryoichi		Hamasuna	Kitakyushu	Japan
Ramin	--	Hakimzadeh	Ormiyeh	Iran
Lars		Haggarth	Stockholm	Sweden
Susanne		Hagel	Berlin	Germany
Marios		Hadjipavlou	London	United Kingdom
Nassya	S.	Hadjieva	Sofia	Bulgaria
Perviz		Haciyev	Ankara	Turkey
Mohamad	Alsayed	Habous	Jedda	Saudi Arabia
U	Sin	Ha	Seoul	Korea, South
Prem	Raj	Gyawali	Kathmandu	Nepal
Laszlo		Gyanyi	Budapest	Hungary

Antonio		Gutierrez	L`Hospitalet	Spain
Necati		Gürbüz	Istanbul	Turkey
Cenk		Gurbuz	Istanbul	Turkey
Alfons		Gunnemann	Detmold	Germany
Adnan		Gündoğdu	İzmir	Turkey
Tawiz	Gul	Gul	Doha	Qatar
Serigne	Magueye	Gueye	Dakar	Senegal
Borut		Gubina	Ljubljana	Slovenia
Angelo		Guarriello	Andria	Italy
JesãƒâºS	Guajardo	Guajardo	Lleida	Spain
Gevorg	V.	Grigoryan	Yerevan	Armenia
Daniel		Grell	Berlin	Germany
Mireille		Gregoire	Quebec	Canada
Francesco		Greco	Halle Saale	Germany
H.J.		Graff	Solingen	Germany
Arthur		Grabsky	Yerevan	Armenia
Beata		Grabowska	Orebro	Sweden
Magnus	J	Grabe	Malmo	Sweden
Hesham	Saad	Gouda	Alexandria	Egypt
Eduardo		Gotuzzo	Lima	Peru
Dmitriy	Gennadievich	Goryainov	Donetsk	Ukraine
Sadik		Gorur	Antakya/Hatay	Turkey
Maximilien		Goris Gbenou	Valence	France
Mehmet Resit		Goren	Ankara	Turkey
Ravisankar		Gopakumarapillai	Manama	Bahrain
Juan	Uria	Gonzalez-Tova	Vic	Spain
Raquel		Gonzalez-Lopez	Madrid	Spain
Sergio	Norbert	Gonzalez-Bittner	Frankfurt	Germany
Francisco		Gomez Veiga	A Coruna	Spain
Yunus	Emre	Göğer	Konya	Turkey
Marcelo	Alvear	Godoy	Santiago	Chile
Alexander	Johannes	Glowik	Hannover	Germany
Bartek		Gliniewicz	Szczecin	Poland
Sidney		Glina	Sao Paulo	Brazil
Jochen		Gleissner	Wuppertal	Germany
Dimitrios		Glaritis	Heraklio	Greece
Canepa		Giorgio	Genoa	Italy
Fernando	J.	Giordano	Merida	Venezuela
Thomas		Gilbert	Koblenz	Germany
Achilles		Gikas	Heraklion	Greece
Vladimir		Giblo	Hradec Kralove	Czech Republic
Claudio	Antonio	Giberti	Chiavari	Italy
Artur		Gibas	Gdansk	Poland
Assem	Sayed-Ahmed	Ghallab	Tanta	Egypt
Sobhan		Ghafouryan	Ilam	Iran
Ardalan	Abdolghafouri	Ghafouri	Doha	Qatar

Ulrich		Gertenbach	Hagen	Germany
Lara		Gerbrandy-Schreuders	Amsterdam	Netherlands
Ventsislav	Dinkov	Georgiev	Ruse	Bulgaria
Georgios		Georgiadis	Redhill	United Kingdom
Andrey	Alexandravich	Gavrusev	Minsk	Belarus
Teresita	Tanaglin	Gaviola	Makati	Philippines
Sandro	Silva	Gaspar	Lisboa	Portugal
Ioannis	Dimitrios	Garifallos	Samos	Greece
Vanessa		Gardikou	Pireas	Greece
Francisco	Antonio	Garcia Velandria	Valencia	Venezuela
Antonio	Sãƒâ¡Nchez	Garcãƒâa	Linares	Spain
Maria		Garabasova	Trnava	Slovakia
Thirumalai		Ganesan	Chennai	India
Carlos		Gamiãƒâ±O	DF	Mexico
Catarina	Diogo	Gameiro	Lisboa	Portugal
Roman	Vladislavovich	Gamazkov	St-Petersburg	Russia
Xavier		Gamãƒâ©	Toulouse	France
David	J	Galvin	Dublin	Ireland
Michele		Gallucci	Rome	Italy
Natalya	Gennagievna	Galkina	Penza	Russia
Carlos	Pacheco	Gahbler	Mexico, D.F.	Mexico
Juan		Gãƒâ³Mez Rivas	Madrid	Spain
Sandor		Gecs	Veszprem	Hungary
Begench	Gurbangeldiyevich	Gadamov	Ashgabat	Turkmenistan
Dr Khaled	Abdul Moneim	Gadalla	Cairo	Egypt
Mauro		Gacci	Florence	Italy
Yaowen		Fu	Changchun	China
Hans-Martin		Fritsche	Regensburg	Germany
Cristina	Cristina	Freuler	Buenos Aires	Argentina
Olga		Fraschini	Lecco	Italy
Michail		Frank	Ekaterinburg	Russia
Duval		Francois	Reims	France
Gianfranco		Formicola	Napoli	Italy
Eleftherios	D	Fokaefs	Patras	Greece
Zoltãn		Florian	Nagykanizsa	Hungary
Christian		Fisang	Bonn	Germany
Juliane		Fiebich	Burgwedel	Germany
Mariaconsiglia		Ferriero	Rome	Italy
Vavassori	Ivano	Ferdinando	Bergamo	Italy
Pal		Fel	Dombovar	Hungary
Michal		Fedorko	Brno	Czech Republic
Gaiseniuk	Z	Fedir	Kiev	Ukraine
Narcisi		Federico	Tordino Teramo	Italy
Zsolt		Fazakas	Budapest	Hungary

Antal	-	Farkas	Debrecen	Hungary
Rui	Almeida	Farinha	Lisbon	Portugal
Yasser	Abd Elraouf	Farahat	Dubai	United Arab Emirates
Bernhard		Fangmeyer	Lingen	Germany
Walid		FALOU	Beirut	Lebanon
Siavash		Falahatkar	Rasht	Iran
Christian	Christian	Fahr	Reutlingen	Germany
Amogu	Kalu	Eziyi	Osogbo	Nigeria
Bakurov	E	Evgeny	Rostov-On-Don	Russia
Fleury		Estelle	Saint Aubin Sur Scie	France
Iouri	M	Essilevski	Moscow	Russia
Khadigeh		Esmaeili	Ilam	Iran
Erkan		Erkan	Istanbul	Turkey
Erbil		Ergenekon	Istanbul	Turkey
Seydali		Eredjepov	Ferghana	Uzbekistan
Erhan	-	Erdogan	Karaman	Turkey
Ibrahim		Erayman	Konya	Turkey
Carmen		Enguita	Madrid	Spain
Christina	Luise	Engels	Karlsruhe	Germany
Marcus		Elstermann Von Elster	Berlin	Germany
Emad	Rashad Mohamed	Elsobky	Abu Dhabi	United Arab Emirates
Alaa	Ali	Elshennawy	Cairo	Egypt
Medhat	Ahmed Mohamed	Elsayed	Abu Dhabi	United Arab Emirates
Maria	Carlsen	Elkjãƒâ¦R	Aarhus	Denmark
Rehab	Hosny	El-Sokkary	Zagazig	Egypt
Claus		Eisenreich	Bad Tolz	Germany
Solymossy		Egon	Székesfehérvár	Hungary
Christian		Eggersmann	Rheine	Germany
Ioannis	Panagiotis	Efthimiou	Chania	Greece
David		Ebralidze	Tbilisi	Georgia
Konstantin	Antonovich	Dunets	Saint-Petersburg	Russia
Athanasius	Daud	Dube	Harare	Zimbabwe
Kim	Maksut	Drasa	Tirana	Albania
Elisaveta	Asenova	Draghijeva	Kuwait	Kuwait
Tomasz		Drabarek	Gdansk	Poland
Elif		Doyuk Kartal	Eskisehir	Turkey
Roland		Donat	Edinburgh	United Kingdom
Andreas	Peter	Dominik	Giessen	Germany
Alexey	Alexeevitch	Dolgiy	Saint-Petersburg	Russia
Razvan	Vasile	Dican	Hof	Germany
Diab	El-Sayed	Diab	Zagazig	Egypt
Manuel		Di Biase	Perugia	Italy

Laura	Frances	Derbyshire	Salford	United Kingdom
Nese		Demirturk	Afyon	Turkey
Erhan		Demirelli	Giresun	Turkey
Umut		Delibas	Istanbul	Turkey
Giulio		Del Popolo	Firenze	Italy
Kojic	Dusan	Dejan	Belgrade	Serbia and Montenegro
Liana	Monica	Deac	Cluj-Napoca	Romania
Geert		De Naeyer	Aalst	Belgium
Jose	Carneiro	De Moura	Lisbon	Portugal
Ferdinando		De Marco	Grottaferrata (Rome)	Italy
Siviardo	Cristobal	De Leon	Zapopan Jalisco	Mexico
Gaetano		De Grande	Siracusa	Italy
Gioacchino		De Giorgi	Udine	Italy
Domenico		De Carolis	Ascoli P	Italy
Mark		Davis	Newcastle Upon Tyne	United Kingdom
John	Brinley	Davies	Newcastle Upon Tyne	United Kingdom
Anton		Dashko	Moscow	Russia
Amitabh		Dash	Delhi	India
Debi	Narayan	Das Chaudhury	PERTH	United Kingdom
Fontana		Dario	Torino	Italy
Ziad		Daoud	Tripoli	Lebanon
Quang Oanh		Dao	Hochiminh	Vietnam
Michael		Dan	Holon	Israel
Le Dinh		Dam	Les Abymes	Guadeloupe
Izak		Dalva	Ankara	Turkey
Hani	R	Dahmash	Der Ezore	Syria
Hugo	Alberto	Dãƒâ¡Vila	Caracas	Venezuela
Xhevdet	M.	Cuni	Prishtina	Albania
Bogdan	A.	Culibrk	Subotica	Serbia and Montenegro
Jose	Angel	Cuesta-Alcalãƒâ¡	Tudela (Navarra)	Spain
Judit		Csorba	Debrecen	Hungary
Cristiano		Cristini	Rome	Italy
Pietro		Cozzupoli	Reggio Calabria	Italy
Pedro		Coteron	Murcia	Spain
Elisabetta		Costantini	Perugia	Italy
Bogdan		Costache	ARAD	Romania
Tiago	Pinto	Correia	Matosinhos	Portugal
Nick		Cooley	London	United Kingdom
Sophie		Conquy	Paris	France
Nicholas		Cohen	Aberdeen	United Kingdom
Manuel	Ferreira	Coelho	Amadora	Portugal

Andrea		Cocci	Firenze	Italy
Liliana	Ofelia	Clara	Buenos Aires	Argentina
Matteo	R.	Ciuffreda	Parma	Italy
Sinharib		Citgez	Istanbul	Turkey
Ettore		Cirillo Marucco	Andria	Italy
Janusz	Andrzej	Ciechan	Lublin	Poland
Izzet		Cicekbilek	Zonguldak	Turkey
Jae Min		Chung	Busan	Korea, South
Hong		Chung	Chungju	Korea, South
Susanne		Christmann	Ludwigshafen	Germany
Michael		Chrisofos	Athens	Greece
Liaqat		Chowoo	Scunthorpe	United Kingdom
Kostas	Nik	Chondros	Heraklion	Greece
Jin-Bong	.	Choi	Gyeonggi-Do	Korea, South
Hyun-Sop		Choe	Suwon	Korea, South
Yong-Hyun		Cho	Seoul	Korea, South
Wonyeol		Cho	Busan	Korea, South
Kang Jun		Cho	Bucheon-City	Korea, South
In-Rae		Cho	Koyang/Gyunggid o	Korea, South
Archil		Chkhotua	Tbilisi	Georgia
Daniel	Mihai	Chirita	Piatra Neamt	Romania
Edmund		Chiong	Singapore	Singapore
Xi		Cheng	Taunton	United Kingdom
Yee-Chun		Chen	Taipei	Taiwan
Ming		Chen	Nanjing	China
Christophe		Chemaslãƒâ€°	Palmerston North	New Zealand
Stavros	N	Charalambous	THESSALONIKI	Greece
Sammy	K K	Chan	Hong Kong	Hong Kong
Suchart		Chaimuangraj	Bangkok	Thailand
Mehmet		Cetinkaya	Istanbul	Turkey
Mete		Çek	Edirne	Turkey
Treuthardt		Cedric	Lausanne	Switzerland
Diego	Martin	Cecchini	Buenos Aires	Argentina
Oğuz	Özden	Cebeci	Ankara	Turkey
Nelson	A.	Cayco	Cabanatuan	Philippines
Luis	Augusto	Castro Sãƒâ¡Der	Barcelona	Spain
Octavio	Augusto	Castillo	Santiago	Chile
Daniele	D	Castellani	Viterbo	Italy
Turhan		Caskurlu	Istanbul	Turkey
Biljana	Luka	Carevic	Belgrade	Serbia and Montenegro
Andrãƒâ©	Martins	Cardoso	Matosinhos	Portugal
Enrique		Cao Avellaneda	Murcia (Murcia)	Spain
Yanwei		Cao	Qingdao	China
Zeynel		Canoğulları	Turkey	Turkey

Josep		Campa	Gasteiz	Spain
Chibelean	Bogdan	Calin	Bucharest	Romania
Emanuele		Caldarera	Palermo	Italy
Tiziana		Calcagno	Genova	Italy
Adriano	Almeida	Calado	Recife	Brazil
Basri		Çakıroğlu	Istanbul	Turkey
Tommaso		Cai	Trento	Italy
Josa	Pedro	Cadilhe	Viana Castelo	Portugal
Susana	Gabriela	Cabrera	Montevideo	Uruguay
Juan	Pablo	Caballero	Alicante	Spain
Xhemil	Ramiz	Bytyci	Prishtina-Kosovo	Serbia and Montenegro
Istvãn		Buzogãny	Budapest	Hungary
Joerg		Busche	Diepholz	Germany
Almudena		Burillo	Madrid	Spain
Stefan		Buntrock	Arendal	Norway
Burak	Besir	Bulut	Kahramanmaras	Turkey
Hubert		BUGEL	ELBEUF	France
Igor		Brzakovic	Novi Sad	Serbia and Montenegro
Franck		Bruyere	Tours	France
Sebastiano		Bruschetta	Messina	Italy
Milos		Brodak	Hradec Kalove	Czech Republic
Oliver		Brock	Hof	Germany
Elena	Vladimirovna	Brizhatyuk	Novosibirsk	Russia
David		Brix	Wuerzburg	Germany
Konstantinos		Bratsas	Haidari, Athens	Greece
Frederico	A.D.	Branco	Porto	Portugal
Tommaso		Brancato	Albano Laziale	Italy
Ugur		Boylu	Istanbul	Turkey
Pierluigi		Bove	Rome	Italy
Philip	S	Bova	Rostov-On-Don	Russia
Andreas		Bourdoumis	Exeter	United Kingdom
Amine		Bouassida	Lens	France
Henry		Botto	Suresnes	France
Nagy		Botond	Brasov	Romania
Francisco	J. Santos	Botelho	Porto	Portugal
Pia	Carla	Bossola	Domodossola	Italy
Ricardo	G.	Borges	Coimbra	Portugal
Gernot		Bonkat	Basel	Switzerland
Giovanni		Bonfiglio	Catania	Italy
Ahmet		Bolukbasi	Izmir	Turkey
Jean-Paul		Boiteux	Clermont-Ferrand	France
Sotirios		Bogris	London	United Kingdom
Csaba		Bognãƒâ¡R	Miskolc	Hungary
Malte		Boehm	Dillenburg	Germany

Katharina		Boehm	Hamburg	Germany
Balazs		Bodrogi	Miskolc	Hungary
Franco		Blefari	Orvieto	Italy
Brian	Penero	Blas	Tacloban, Leyte	Philippines
Truls	Erik	Bjerklund Johansen	Oslo	Norway
Jure		Bizjak	Ljubljana	Slovenia
Werner	H	Bischoff	Backnang	Germany
Rasmus		Bisbjerg	Herlev	Denmark
Brian	Robert	Birch	Southampton	United Kingdom
Renu		Bharadwaj	Pune	India
Aashutosh		Bhanot	Pleven	Bulgaria
Shashikant	R	Bhange	Uttam Plaza, Pune	India
Burkhard		Beyer	Hamburg	Germany
Ingrid	H	Berger	Wien	Austria
Boris	Vitalyevitch	Berejanski	Moscow	Russia
Eduardo		Bercowsky	Menorca	Spain
Saida		Benredjeb	Tunis	Tunisia
Francesco		Beniamin	Treviso	Italy
Giuseppe		Benedetto	Padova	Italy
Nawfel		Ben Rais	Tunis	Tunisia
Payam		BEHZADI	Tehran	Iran
Selahattin		Bedir	Ankara	Turkey
Jaroslav		Beck	Kosice	Slovakia
Pastora		Beardo	Jerez De La Frontera	Spain
Mustafa		Bazardzanovic	Tuzla	Bosnia and Herzegovina
Rıza		Baysal	Antalya	Turkey
Yildirim		Bayazit	Adana	Turkey
Mihir	V	Baxi	Mumbai	India
Michael		Baumann	Hameln	Germany
Stojan		Batandjiovski	Bitola	Macedonia, The Former Yugoslav Republic of
Paulo	Roberto	Bastos	Niteroi Rio De Janeiro	Brazil
Ralf	F.	Basting	Altoetting	Germany
Harry		Bassaris	Patras	Greece
Gediminas		Baseckas	Ystad	Sweden
Riccardo		Bartoletti	Pistoia	Italy
Joeri		Barth	Ghent	Belgium
Lyidmila	Pavlovna	Barashova	Kemerovo	Russia
Caner		BARAN	Kahramanmaras	Turkey
Firuz		Barakaev	Moscow	Russia
Maria	Del Carmen	Bangher	Corrientes	Argentina

Vladimir	Mihailo	Bancevic	Belgrade	Serbia and Montenegro
Goran	Krume	Balevski	Ohrid	Macedonia, The Former Yugoslav Republic of
Melih		Balci	Ankara	Turkey
Mehmet		Balasar	Konya	Turkey
Mohammad		Bakhtiar	London	United Kingdom
Senad		Bajramovic	Sarajevo	Bosnia and Herzegovina
Gabor		Bajor	Budapest	Hungary
Seung		Baik	Gwang-Ju	Korea, South
Badrulhisham		Bahadzor	Cheras, KL	Malaysia
Mohamed	Alaa	Bahaa	Nyala	Sudan
Mahmood	Reza	Baghinia	Arak	Iran
Marcos	Alessandro	Baeza	Umuarama Pr	Brazil
Jae Hyun		Bae	Ansan	Korea, South
Cãƒâ©Cile		BACH	Suresnes	France
Birgit	Kaa	Bach	Aarhus N	Denmark
Abdul	Azim	Azizi	Herlev	Denmark
Olgun		Azis	Constanta	Romania
Behrooz	Rahnavardi	Azari	Bandar Abbas	Iran
Omer		Aytac	Istanbul	Turkey
Serdar		Aykan	Istanbul	Turkey
Elin		Axen	Gothenburg	Sweden
M Hammad		Ather	Karachi	Pakistan
Grigorios		Athanasiadis	Guildford	United Kingdom
Muhammad		ATFA	Aulnay Sous Bois	France
Ferhat		Ates	Istanbul	Turkey
Henry	None	Atawurah	Takoradi	Ghana
Rasul	Akhmedjanovich	Ataniyazov	Tashknet	Uzbekistan
Ali		Atan	Ankara	Turkey
Mehmet	Kazim	Asutay	Istanbul	Turkey
Mohamadali		Aslmonadi	Tabriz	Iran
Yilmaz		Aslan	Ankara	Turkey
Ergul		Aslan	Istanbul	Turkey
Ahmet	Ruknettin	Aslan	Istanbul	Turkey
Ramazan		Asci	Samsun	Turkey
Cagri		Asan	BATMAN	Turkey
Montserrat		Arzoz	Badalona	Spain
Levon	Dm	Arustamov	Tashkent	Uzbekistan
Burak		Arslan	Istanbul	Turkey
Nasr		Arsanious	Croydon	United Kingdom
Szanto		Arpad	Pecs	Hungary
Athanasios	N	Argyropoulos	Elefsina	Greece

Manuel	Alvarez	Ardura	Alcorcon, Madrid	Spain
Ersan		Arda	Edirne	Turkey
Alejandro		Arce	Mexico	Mexico
Saeid		Arasteh	Bam	Iran
Haykaz		Antonyan	Yerevan	Armenia
Abi Aad		Antoine	Brussels	Belgium
Javier		Angulo	Getafe, Madrid	Spain
Catarina	Ingrid	Aneman	Goteborg	Sweden
Alexander	Vladimirovich	Andreychikov	Krasnoyarsk	Russia
Elena		Andretta	Dolo (Venice)	Italy
Kim	Hovgaard	Andreassen	Fredericia	Denmark
Anders	Christian	Andersson	Limhamn	Sweden
Leif	Percival	Andersen	Copenhagen Ãƒëœ	Denmark
Jens	Thorup	Andersen	Roskilde	Denmark
Polvonov	Abror	Aminovich	Samarkand	Uzbekistan
Miguel		Alvarez-Mugica	Oviedo	Spain
S B Pavan Kumar		Aluru	Sunderland	United Kingdom
Muammer		Altok	Izmir	Turkey
Mustafa		Altindis	Sakarya	Turkey
Javier	David	Altclas	Buenos Aires	Argentina
Nayel	Abdullah	Altarawneh	Doha	Qatar
Berthold		Alt	Fulda	Germany
Naimet	Kamal	Alsaigh	Alain - Abudhabi	United Arab Emirates
Abdulmunem	Mohammed	Alsadi	Dubai	United Arab Emirates
Caner		Alptekin	Izmir	Turkey
Ahmed	Mabrouk	Alosta	London	United Kingdom
Vafa	Abd	Allahpour	Mahabad	Iran
Ahmad	Ashhab	Alkadi	Ryiad	Saudi Arabia
Ahmed	Khalil	Aljubory	Mosul	Iraq
Jakhongir	Fatikhovich	Alidjanov	Tashkent	Uzbekistan
Tarek		Ali	Nyiregyhaza	Hungary
Arif	Maqsood	Ali	Okara	Pakistan
Ahmed	Ragab	Ali	Sharkia	Egypt
Ammar		Alhasso	Dunfermline	United Kingdom
Gamal	Mohammed Sami	Alhadad	Alexandria	Egypt
Naser	Abdussalam	Alfgi	Tripoli	Libya
Shulyak		Alexander	Lviv	Ukraine
Egote	Kofi	Alexander	Moscow	Russia
Dmitry		Aleshin	Brno	Czech Republic
Mohammad	Khalil Ibrahim	Aldahiri	Abu Dhabi	United Arab Emirates
Emilio	Lopez	Alcina	Valencia	Spain
Istvan		Albert	Budapest	Hungary
Zaheer		Alam	Karachi	Pakistan
Bulent		ALAGOL	Edirne	Turkey

Muna	Mohsen	Al-Haidary	Hodeidah	Yemen
Kais		Al-Dairi	Chelmsford	United Kingdom
Nahed	Ahmad	Al Tabash	Taif	Saudi Arabia
Riyadh		Al Salh	Dammam	Saudi Arabia
Ilker	-	Akyol	Istanbul	Turkey
Sergey	Dmitrievich	Aksionov	Kiev	Ukraine
Yigit		Akin	Antalya	Turkey
Waseem		Akhter	London	United Kingdom
Hacã„Â±	Murat	Akgãƒâ¼L	Istanbul	Turkey
Ali	Ferruh	Akay	Istnbul	Turkey
Ulrike		Ahrens	Ulm	Germany
Jae Hyun		Ahn	Yangsan-Si, Gyeongnam	Korea, South
Mumtaz		Ahmad	Rawalpindi	Pakistan
Christer		Ahlstrand	Linköping	Sweden
Reza		Aghelnezhad	Mashhad	Iran
Mehmet		Oã„Å¸Lu	Gaziantep	Turkey
Akisibadek	Alekz	Afoko	Tamale	Ghana
Vasileios	Georgios	Adamopoulos	Heraklio	Greece
Przemyslaw		Adamczyk	Torun	Poland
Iwan	Asmara	Achmad	Jakarta	Indonesia
Kamal	Jamil	Achkar	Creil	France
Ömer		Acar	Istanbul	Turkey
Refaat	Mohamad	Abusamra	Misrata	Libya
Tarik	Sulaiman	Abulhul	Tripoli	Libya
Salisu		Abubakar	Kano	Nigeria
Leif		Abramsson	Umeãƒâ¥	Sweden
Ernesto	A	Aboytes	Celaya	Mexico
Vitaly	Eduardovich	Aboyan	Rostov-On-Don	Russia
Mohamed		Abo El-Enen	Tanta	Egypt
Mohamed	Ismat	Abdulmajed	Milton Keynes	United Kingdom
Iskander	Ilfakovich	Abdullin	Moscow	Russia
Muthanna	Saad	Abduljawad	Mosul	Iraq
Bouzouita		Abderrazak	Tunis	Tunisia
Amr	Mahmoud	Abdelhakim	Cairo	Egypt
Khaled	Farid	Abdel-Aziz	Dallas	United States
Hatem	Mahrous	Abdel Wahab	Cairo	Egypt
Antonino		Abbolito	Roma	Italy
Bruno		Abbate	Florence	Italy
Malashchitsky	Dmitry	A.	Minsk	Belarus
